# Localized COUP‐TFII pDNA Delivery Modulates Stem/Progenitor Cell Differentiation to Enhance Endothelialization and Inhibit Calcification of Decellularized Allografts

**DOI:** 10.1002/advs.202409744

**Published:** 2024-12-10

**Authors:** Mengmeng Xing, Fei Wang, Ruowen Chu, He Wang, Yuyao Sun, Meng Qian, Huan Jiang, Adam C. Midgley, Guohao Dai, Qiang Zhao

**Affiliations:** ^1^ State key Laboratory of Medicinal Chemical Biology Frontiers Science Center for Cell Responses Key Laboratory of Bioactive Materials (Ministry of Education), College of Life Sciences Nankai University Tianjin 300071 China; ^2^ Department of Bioengineering Northeastern University Boston MA 02115 USA; ^3^ The Institute of Cardiovascular Sciences, School of Basic Medical Sciences, State Key Laboratory of Vascular Homeostasis and Remodeling Health Science Center Peking University Beijing 100191 China

**Keywords:** calcification, COUP‐TFII, decellularized allografts, endothelial differentiation, stem/progenitor cells (SPCs)

## Abstract

Decellularized allografts have emerged as promising candidates for vascular bypass grafting, owing to their inherent bioactivity and minimal immunogenicity. However, graft failure that results from suboptimal regeneration and pathological remodeling has hindered their clinical adoption. Recent advances in vascular biology highlight the pivotal role of COUP‐TFII in orchestrating endothelial identity, angiogenesis, safeguarding against atherosclerosis, and mitigating vascular calcification. Here, plasmid DNA (pDNA) encoding COUP‐TFII is incorporated into decellularized allografts to realize localized delivery. Comprehensive in vitro investigation complemented by a bone marrow transplantation model on genetic‐lineage‐tracing mouse revealed the underlying mechanisms of COUP‐TFII in regulating vascular regeneration and remodeling. COUP‐TFII augmented endothelialization and inhibited calcification in decellularized allografts by modulating the Ang1/Tie2/PI3K/AKT signaling pathway that dictates the fate of Sca‐1^+^ stem/progenitor cells. Heparin‐polyethyleneimine nanoparticles (HEPI) are prepared as COUP‐TFII pDNA nanocarriers (COUP‐TFII@HPEI) and used to modify decellularized allografts, achieving long‐term and stable overexpression of COUP‐TFII. Functionalized grafts are evaluated in rat abdominal artery replacement models, demonstrating enhanced neo‐artery regeneration without calcification. The study provides an effective strategy to enhance the applicability of decellularized allograft and illustrates their translational prospects for vascular bypass grafting.

## Graphical abstract

COUP‐TFII is a venous transcription factor that play pivotal roles in vascular regeneration and remodeling. A decellularized allograft functionalized with COUP‐TFII@HPEI is prepared. Localized COUP‐TFII pDNA delivery from HEPI nanocarriers efficiently promotes vascular regeneration and attenuates vascular calcification. Mechanistically, COUP‐TFII promotes the differentiation of resident stem/progenitor cells toward endothelial lineage via activating the Ang1/Tie2/PI3K/AKT signaling pathway.

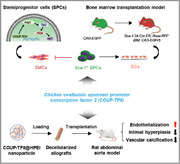

## 1 Introduction

Cardiovascular diseases (CVDs) remain the leading cause for high morbidity and mortality in the world. In 2022, an estimated 330 million patients were burdened by CVDs, which account for approximately 48.00% and 45.86% of the causes of death in rural and urban areas, respectively.^[^ ^1^ ^]^ Cardiovascular defects, injuries, and degenerative diseases such as coronary heart disease, heart failure, and stroke often require vascular interventions.^[^ ^2^ ^]^ Such surgical procedures involve a high demand for vascular grafts. However, currently utilized traditional grafts face problems with thrombogenicity, susceptibility to infection, and poor long‐term patency rates.^[^ ^3^, ^4^, ^5^ ^]^ Decellularization of native blood vessels (arteries and veins) offer a promising alternative, benefiting from inherent bioactivity that can be more readily accepted, integrated, and remodeled by host tissue after implantation, but often encounter problems associated with adverse remodeling, including intimal hyperplasia and calcification.^[^ ^6^, ^7^ ^]^ Accumulating evidence has established that functional modification of vascular grafts with vasoactive substances can elicit specific biological functions that favor tissue regeneration and remodeling, thus long‐term patency could be anticipated.^[^ ^7^, ^8^, ^9^, ^10^, ^11^, ^12^, ^13^, ^14^ ^]^

Rapid re‐cellularization,^[^ ^15^, ^16^ ^]^ restoration of a functional endothelium,^[^ ^17^, ^18^, ^19^ ^]^ and minimal intimal hyperplasia^[^ ^20^, ^21^ ^]^ are essential for long‐term graft performance. Recent studies reported that vascular resident stem/progenitor cells (SPCs) play important roles in vascular homeostasis and remodeling.^[^ ^17^, ^22^ ^]^ The adventitia of aortic roots contained a large number of cells expressing stem cell markers, e.g., Sca‐1^+^ (21%), c‐kit^+^ (9%), CD34^+^ (15%), and Flk1^+^ cells (4%).^[^ ^23^ ^]^ Among these, the adventitial Sca‐1^+^ cells possess the potential to differentiate into different vascular cell lineages, including endothelial cells (ECs) and smooth muscle cells (SMCs),^[^ ^24^, ^25^, ^26^ ^]^ and play roles in endothelialization, regeneration of smooth muscle layers, and immunomodulation.^[^ ^27^ ^]^ These actions have been shown to lead to an orchestrated cellular response that promotes vascular regeneration of implanted grafts.

Chicken ovalbumin upstream promoter transcription factor 2 (COUP‐TFII; NR2F2) is a ubiquitously expressed nuclear receptor belonging to the steroid receptor superfamily^[^ ^28^, ^29^ ^]^ with important roles in regulating tissue function,^[^ ^30^, ^31^ ^]^ stem cell differentiation,^[^ ^32^, ^33^ ^]^ angiogenesis,^[^ ^34^, ^35^ ^]^ metabolic homeostasis,^[^ ^31^, ^36^ ^]^ and organ development.^[^ ^34^, ^37^ ^]^ Although expressed in arterial ECs, COUP‐TFII is more highly expressed in venous ECs, wherein it inhibits Notch signaling to guide vessel characteristics toward venous vasculature.^[^ ^38^, ^39^, ^40^ ^]^ We previously showed that the suppression of COUP‐TFII in vascular ECs up‐regulated pro‐inflammatory and artery‐related gene expression while simultaneously impairing the antithrombotic gene expression.^[^ ^41^ ^]^ In addition, inhibited COUP‐TFII promoted osteogenic gene expression in ECs, leading to osteoblast‐like differentiation and calcium deposition.^[^ ^41^ ^]^ A study by the Koh lab demonstrated that over‐expression of COUP‐TFII inhibited BMP2‐mediated osteogenic differentiation in osteoblasts.^[^ ^42^ ^]^ Furthermore, Koh et al. discovered that COUP‐TFII negatively regulated osteoblast differentiation via interaction with Runx2, and BMP2‐induced Runx2 repressed COUP‐TFII expression and promoted osteoblast differentiation during the differentiation state.^[^ ^43^ ^]^ Therefore, COUP‐TFII appears to have protective roles against pathogenic phenotypes resembling atherosclerosis and vascular calcification.

Herein, we took inspiration from the biological functions of COUP‐TFII to design and fabricate a functional vascular graft by combining decellularized allogeneic grafts with plasmid DNA (pDNA) encoding COUP‐TFII. First, both in vitro and in vivo studies indicated that over‐expression of COUP‐TFII effectively promoted endothelial differentiation of Sca‐1^+^ SPCs. In addition, a bone marrow transplantation model in *Sca‐1 2A‐CreER; Rosa‐RFP* genetic‐lineage‐tracing mice provided further insights into the origin and fate of Sca‐1^+^ SPCs in the context of vascular regeneration and remodeling. We then prepared heparin‐polyethyleneimine (HPEI) nanoparticles as carriers of COUP‐TFII pDNA for functionalization of decellularized allografts, which were then systematically evaluated in a rat abdominal aorta replacement model.

## 2 Results

### 2.1 COUP‐TFII Promotes the Differentiation of Sca‐1^+^ Stem/Progenitor Cells Toward Endothelial Lineage via Activating the Ang1/Tie2/PI3K/AKT Signaling Pathway

**Figure 1:**
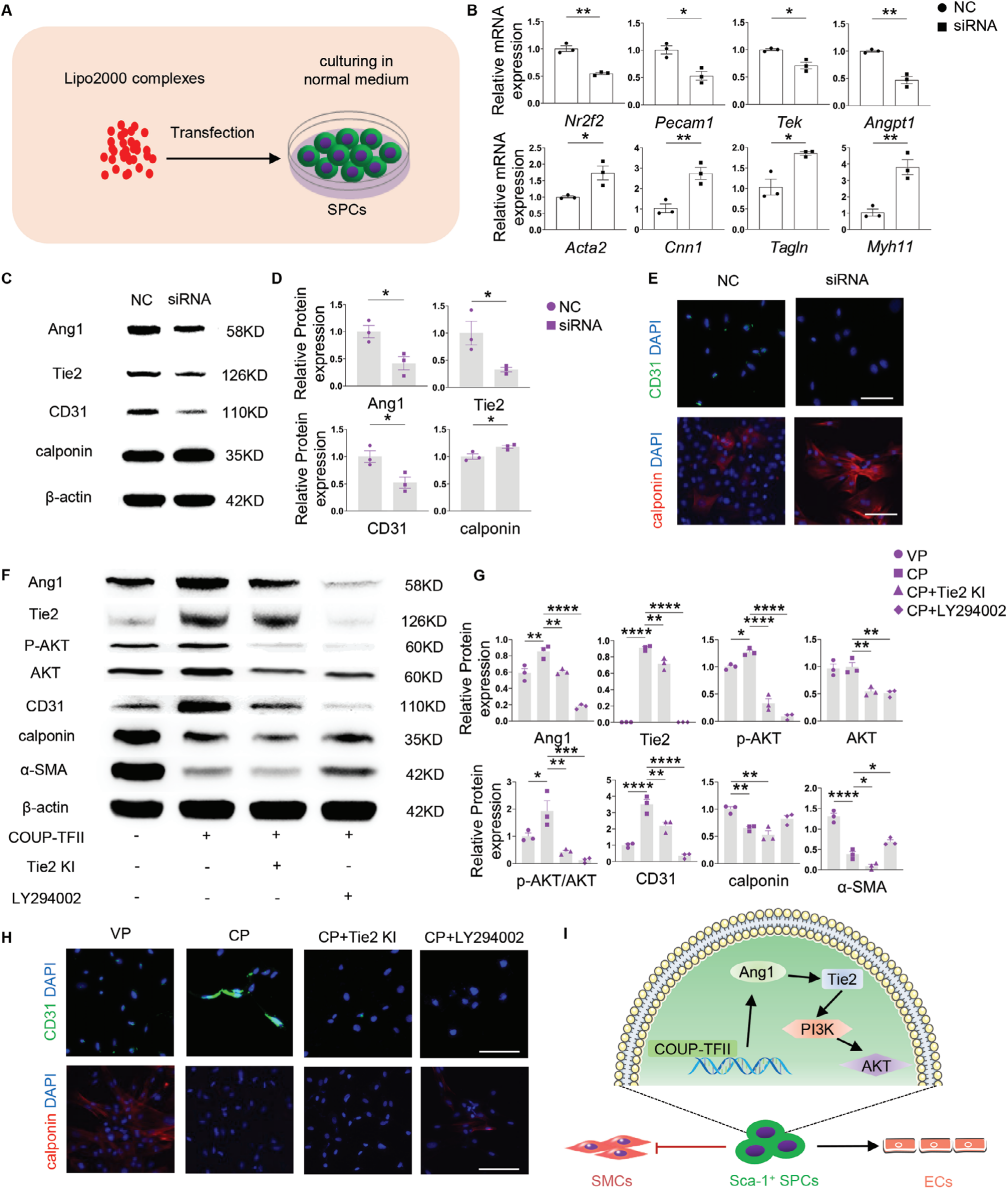
COUP‐TFII regulates Sca‐1^+^ stem/progenitor cells differentiation via activating the Ang1/Tie2/PI3K/AKT signaling pathway. A) Schematic illustration shows the transfection of Sca‐1^+^ SPCs with specific siRNA or plasmid of COUP‐TFII. B) Real‐time qPCR analysis of the Sca‐1^+^ SPCs after different treatments is shown (*n* = 3). C,D) Shown is a Western blot and quantitative analysis of the Sca‐1^+^ SPCs (*n* = 3). E) Immunofluorescence staining for CD31 and calponin of the Sca‐1^+^ SPCs are shown. Scale bar: 50 µm. F,G) Shown is a Western blot and quantification for expression of proteins involved in the Ang1/Tie2/PI3K/AKT signaling pathway after different treatments (*n* = 3). H) Immunofluorescence staining for CD31 and calponin are shown. Scale bar: 50 µm. I) Diagram summarizes the modulatory effect of COUP‐TFII on Sca‐1^+^ SPCs differentiation. All data are presented as the means ± SEM. *p < 0.05, **p < 0.01, ***p < 0.001, ****p < 0.0001. NC: negative control; siRNA: COUP‐TFII siRNA; VP: vacant pDNA; CP: COUP‐TFII pDNA.

Sca‐1^+^ SPCs were isolated from the adventitial of rat aorta and cultured in standard stem cell culturing medium supplemented with leukemia inhibitory factor (LIF), and it was observed that the Sca‐1^+^ SPCs could spontaneously differentiate toward the SMC lineage when LIF was removed from the medium. Previous studies have shown that the expression of Angiopoietin‐1 (Ang1), which mediates mesenchymal–endothelial cell interactions during heart development and vascular remodeling, is down‐regulated in COUP‐TFII mutants.^[^ ^34^ ^]^ Hence, it is reasonable to hypothesize that COUP‐TFII may also play a key role in modulating SPCs commitment and differentiation. To test this hypothesis, COUP‐TFII–specific siRNA was transfected into isolated Sca‐1^+^ SPCs and cultured in normal DMEM medium (**Figure** **1**A). Data obtained from RT‐qPCR indicated that knockdown of COUP‐TFII mRNA (*Nr2f2*) also resulted in the decreased expression of genes associated with ECs, including *Pecam1*, *Tek,* and *Angpt1*. Meanwhile, *Nr2f2* knockdown significantly (*p* < 0.01 or 0.05) upregulated the expression of genes associated with SMCs, including *Acta2*, *Cnn1*, *Tagln*, and *Myh11* (Figure 1B). Further analysis at the protein level, as demonstrated by immunoblotting and immunofluorescence staining, revealed down‐regulated Ang1, Tie2, and CD31, and upregulated calponin (Figure 1C–E). These data established that COUP‐TFII expression is critical to the promotion of SPCs differentiation toward ECs while inhibiting their differentiation toward the SMC lineage.

To further elucidate the function of the COUP‐TFII, pDNA encoding COUP‐TFII was transfected into Sca‐1^+^ SPCs. Over‐expression of COUP‐TFII resulted in the up‐regulation of proteins related to ECs, including Ang1, Tie2, activated AKT (p‐AKT/AKT), and CD31, but also the down‐regulation of proteins associated with SMCs, including calponin and α‐SMA. Activation of AKT was inhibited by the addition of Tie2 inhibitor (Tie2 KI) and PI3K inhibitor (LY294002), which resulted in the abolishment of COUP‐TFII‐induced CD31^+^ ECs differentiation. Notably, the addition of PI3K inhibitor reversed the downregulation of α‐SMA expression (Figure 1F,G). Immunofluorescence staining (Figure 1H) and flow cytometric assays (Figure S1, Supporting Information) further supported the results, which when taken together, suggested that COUP‐TFII promoted the differentiation of the Sca‐1^+^ SPCs toward EC lineage, dependent on the activation of the Ang1/Tie2/PI3K/AKT signaling pathway (Figure 1I).

### 2.2 COUP‐TFII Enhances Endothelialization and Attenuates Calcification of Decellularized Allografts in Mice

**Figure 2:**
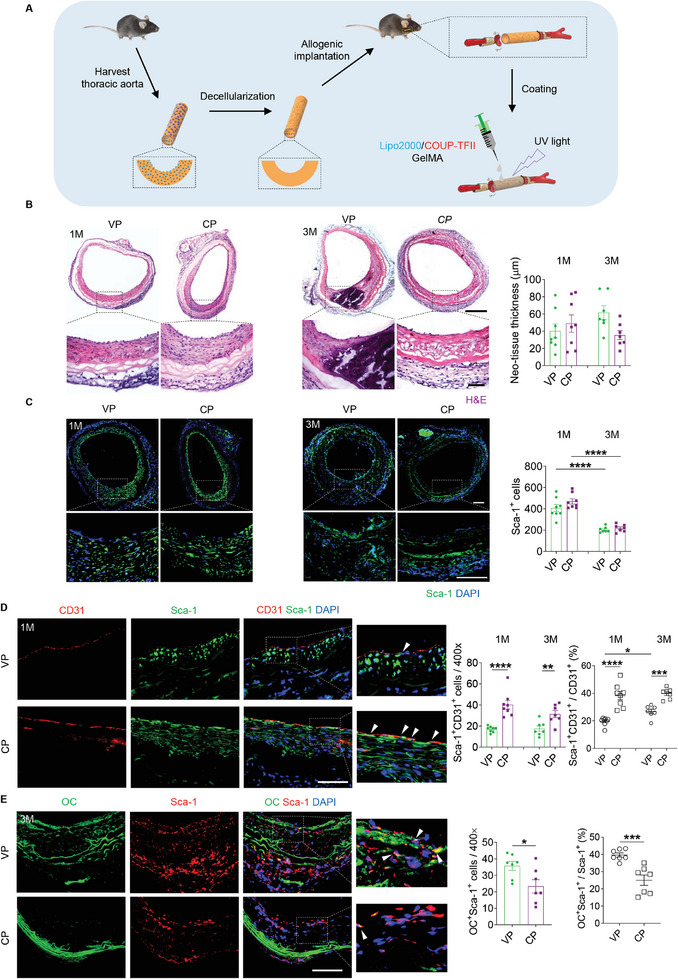
COUP‐TFII overexpression enhances endothelialization and attenuates calcification of decellularized allografts in a mouse model. A) Schematic illustration shows the preparation and implantation of decellularized allografts functionalized with COUP‐TFII pDNA in a mouse carotid artery replacement model. B) H&E staining of the grafts explanted at 1 and 3 months is shown, respectively. Scale bars: 200 µm or 50 µm (magnified images). The thickness of neo‐tissue in two groups was further quantified (*n* = 8 for 1 month; *n* = 7 for 3 months). C) Immunofluorescence staining for Sca‐1 is shown. The number of Sca‐1^+^ cells was quantified (*n* = 8 for 1 month; *n* = 7 for 3 months). Scale bar: 100 or 50 µm (magnified images). D) Co‐immunofluorescence staining for CD31 and Sca‐1 of grafts is shown. Scale bar: 50 µm. The number of Sca‐1^+^CD31^+^ cells and the ratio of Sca‐1^+^CD31^+^ to CD31^+^ cells were quantified (*n* = 8 for 1 month; *n* = 7 for 3 months). E) Co‐immunofluorescence staining for OC and Sca‐1 is shown. Scale bar: 50 µm. The number of OC^+^Sca‐1^+^ cells and the ratio of OC^+^Sca‐1^+^ to Sca‐1^+^ cells were quantified (*n* = 7). White arrows indicate the differentiated SPCs. All data are presented as the means ± SEM. *p < 0.05, **p < 0.01, ***p < 0.001, ****p < 0.0001.

Decellularized allografts loaded with COUP‐TFII‐eGFP pDNA were evaluated in a mouse model of carotid artery replacement (**Figure** **2**A; Figure S2A,B, Supporting Information). The patency of the implanted grafts was first evaluated by Doppler ultrasound (Figure S2C, Supporting Information). Both groups of VP (Vacant‐eGFP pDNA) and CP (COUP‐TFII‐eGFP pDNA) maintained high patency rates after 1 and 3 months of implantation (Figure S2D, Supporting Information). Following graft explantation, stereoscopic images showed that the luminal surfaces were uniformly smooth, with no evidence of thrombosis (Figure S2E, Supporting Information). The neointimal thickness of the two groups was similar in 1 month, as demonstrated by H&E staining (Figure 2B). By 3 months, the neointimal thickness of CP group is smaller than the VP group. Immunofluorescence staining (Figure 2C) indicated that the number of the Sca‐1^+^ SPCs had no significant differences between both groups, but both tended to decrease over time. Co‐immunofluorescence staining for GFP and Sca‐1 further revealed that almost 30% of the Sca‐1^+^ SPCs have been successfully transfected to express COUP‐TFII at 1 month but decreased over time (Figure S3, Supporting Information).

Endothelialization of the vascular grafts was first assessed by immunofluorescence staining for CD31, followed by quantitative analysis, which further confirmed enhanced endothelialization in CP grafts (Figure S4A–C, Supporting Information). SEM observation showed that the cells that were adhered on the luminal surface of CP grafts demonstrated a cobblestone pattern with high coverage and oriented in the direction of blood flow, in contrast to those in the VP groups (Figure S4D, Supporting Information). Importantly, there was an abundance of ECs in the CP grafts that also expressed endothelial NO synthase (eNOS) and VE‐cadherin (CD144), suggesting that COUP‐TFII accelerated the endothelialization process while promoting a mature and functional endothelial phenotype (Figure S4E,F, Supporting Information). After 1 and 3 months of implantation, co‐immunofluorescence staining for CD31 and Sca‐1 validated that COUP‐TFII promoted the differentiation of Sca‐1^+^ SPCs into ECs to achieve endothelialization (Figure 2D). Furthermore, co‐immunofluorescence staining for CD31, GFP, and Sca‐1 uncovered that the transfection of COUP‐TFII into Sca‐1^+^ SPCs did reliably promote the differentiation into ECs (Figure S5, Supporting Information).

The incidence of vascular calcification in the grafts was first assessed by Von Kossa and Alizarin Red staining, which supported that COUP‐TFII attenuated calcification (Figure S6A, Supporting Information). These data were further confirmed by immunofluorescence staining for osteocalcin (OC) (Figure S6B, Supporting Information). Additionally, co‐staining for Sca‐1 and OC indicated that COUP‐TFII attenuated calcification by reducing the number of osteoblast‐like cells that trans‐differentiated from Sca‐1^+^ SPCs (Figure 2E). In addition, the evaluation of the inflammation in the vascular grafts showed that COUP‐TFII increased the presence of M2 macrophages whilst reducing M1 macrophages in the grafts, suggesting a beneficial effect on regulation of inflammatory response after graft implantation (Figure S7, Supporting Information).

### 2.3 The Source of Cells Infiltrating the Decellularized Allografts was Investigated by a Bone Marrow Transplantation Model

**Figure 3:**
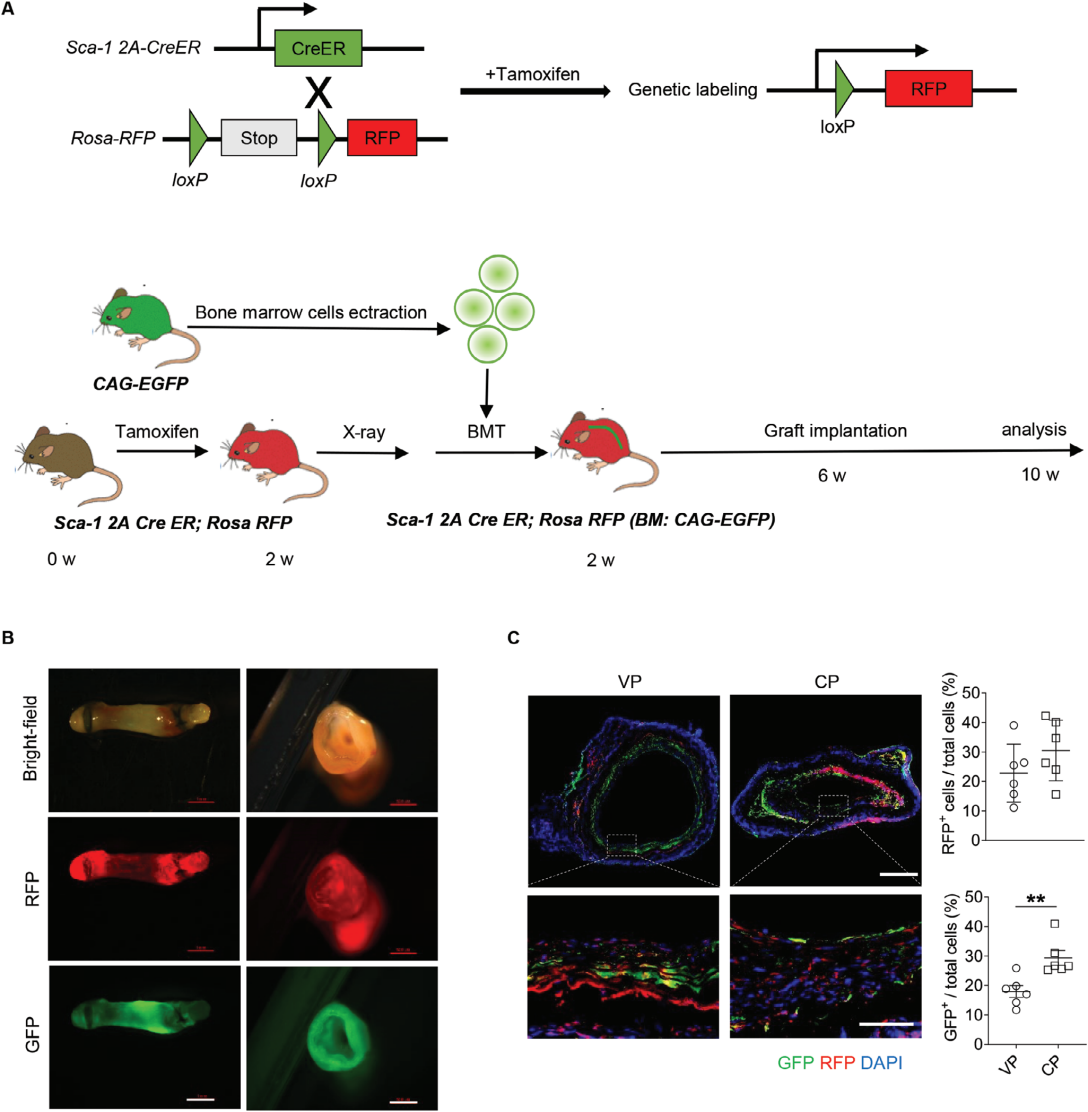
The source of cells repopulating the decellularized allografts was investigated by a bone marrow transplantation model. A) Experimental design shows a bone marrow transplantation model was constructed by transplanting EGFP^+^ bone marrow cells into a *Sca‐1 2A‐CreER; Rosa‐RFP* lineage tracing mouse. B) Stereoscopic images of the explanted grafts are shown. Scale bars: 1 mm (Left) or 500 µm (Right). C) Co‐immunofluorescence staining for GFP and RFP is shown. The ratios of RFP^+^ cells to total cells as well as GFP^+^ cells to total cells were further quantified (*n* = 6). Scale bars: 200 or 50 µm (magnified images). All data are presented as the means ± SEM. **p < 0.01.

To investigate the source of cells repopulating and regenerating the grafts, reconstituted bone marrow (BM) chimera mouse models were established (**Figure** **3**A), and pDNA encoding COUP‐TFII was used (lacking eGFP label; Figure S8A, Supporting Information). BM from CAG‐EGFP mice were transplanted into *Sca‐1 2A‐CreER; Rosa‐RFP* lineage‐tracing mice, wherein resident Sca‐1^+^ SPCs were labeled by *Sca‐1‐CreER* and express RFP when treated with tamoxifen, and cells derived from BM expressed EGFP. Prior to implantation, flow cytometry was used to assess the bone marrow reconstruction rate after transplantation, and only the mice with a reconstruction efficiency of over 90% were selected for use in carotid artery replacement models (Figure S8B, Supporting Information).

Stereoscopic images of the graft explanted at 1 month revealed that both the resident SPCs (RFP^+^ cells) and BM‐derived cells (EGFP^+^ cells) participated in vascular regeneration (Figure 3B). Fluorescence microscopy to visualize GFP^+^ and RFP^+^ cells further verified the involvement of the cells from both origins and that COUP‐TFII promoted the two types of cells to participate in vascular regeneration (Figure 3C).

### 2.4 Both Bone Marrow‐Derived Cells and Resident Stem/Progenitor Cells Contribute to the Vascular Regeneration of Decellularized Allografts

**Figure 4:**
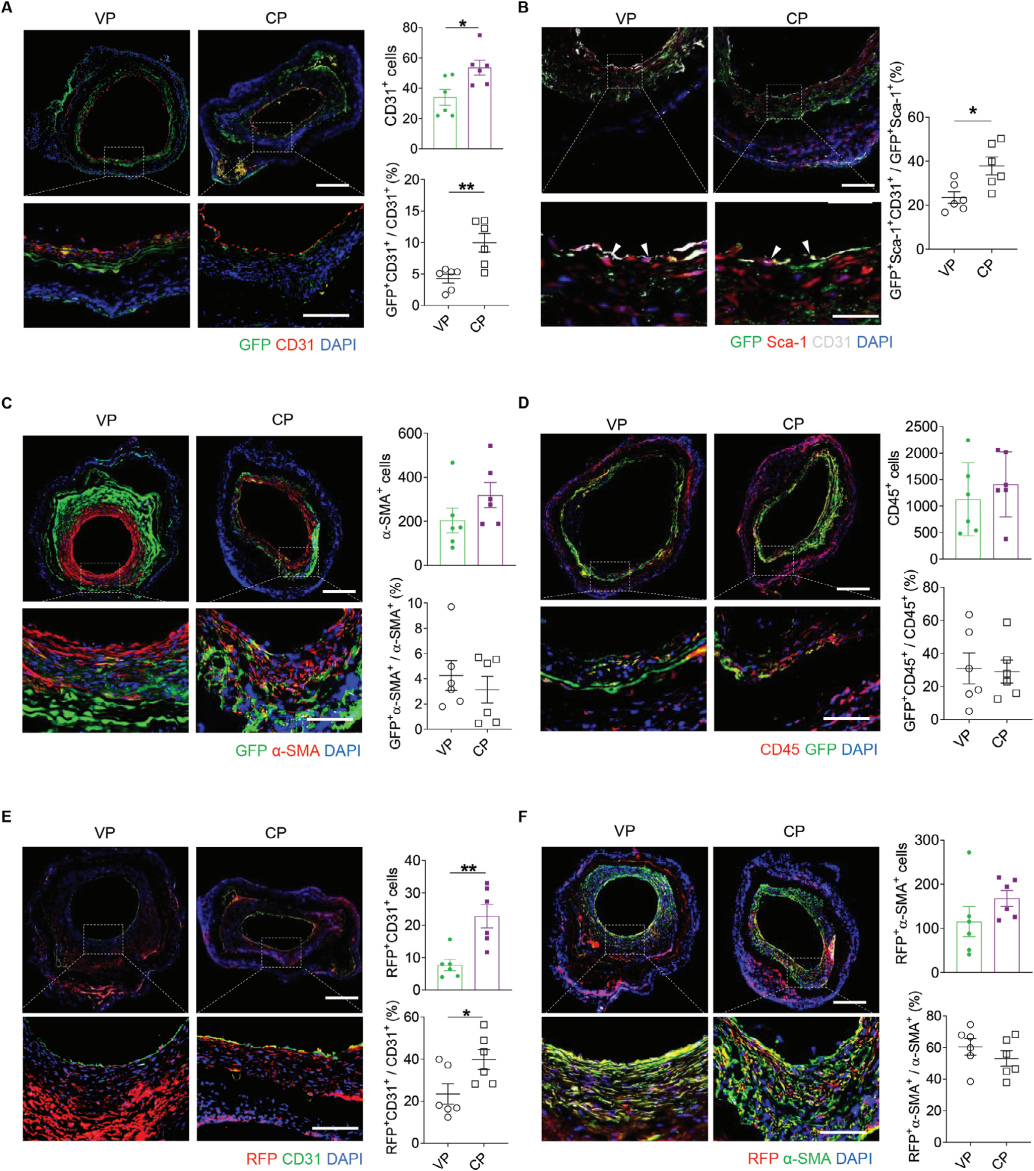
Bone marrow‐derived cells predominantly contribute to ECs and inflammatory cells, while resident stem/progenitor cells mainly contribute to EC and SMC population. A) Co‐immunofluorescence staining for GFP and CD31 is shown. The number of CD31^+^ cells and the ratio of GFP^+^ CD31^+^ cells to CD31^+^ cells were quantified (*n* = 6). Scale bars: 200 or 50 µm (magnified images). B) Co‐immunofluorescence staining for GFP, Sca‐1, and CD31 is shown. Scale bars: 200 or 50 µm (magnified images). White arrows indicate the differentiated SPCs. The ratio of GFP^+^Sca‐1^+^CD31^+^ cells to GFP^+^Sca‐1^+^ cells was further quantified (*n* = 6). C) Co‐immunofluorescence staining for GFP and α‐SMA is shown. The number of α‐SMA^+^ cells and the ratio of GFP^+^α‐SMA^+^ cells to α‐SMA^+^ were quantified (*n* = 6). Scale bars: 200 or 50 µm (magnified images). D) Co‐immunofluorescence staining for GFP and CD45 is shown. The number of GFP^+^CD45^+^ cells and the ratio of GFP^+^CD45^+^ cells to GFP^+^ cells were quantified (*n* = 6). Scale bars: 200 or 50 µm (magnified images). E) Co‐immunofluorescence staining for RFP and CD31 is shown. The number of RFP^+^CD31^+^ cells and the ratio of RFP^+^CD31^+^ cells to CD31^+^ cells were quantified (*n* = 6). Scale bars: 200 or 50 µm (magnified images). F) Co‐immunofluorescence staining for RFP and α‐SMA is shown. The number of RFP^+^α‐SMA^+^ cells and the ratio of RFP^+^α‐SMA^+^ cells to α‐SMA^+^ cells were quantified (*n* = 6). Scale bars: 200 or 50 µm (magnified images). All data are presented as the means ± SEM. *p < 0.05, **p < 0.01.

We next sought to investigate the fate of the BM‐derived cells and resident SPCs and their contribution to the vascular regeneration of the grafts. Co‐immunofluorescence staining for GFP and CD31 proved that whilst COUP‐TFII could drive endothelial differentiation of BM‐derived cells, only a low percentage of GFP^+^CD31^+^ cells (VP: 4.27% ± 0.71% vs CP: 9.95% ± 1.47%) were observable (**Figure** **4**A). Imaging of GFP, Sca‐1, and CD31 co‐expression further indicated that COUP‐TFII promoted the endothelial differentiation of Sca‐1^+^ cells from BM into ECs (Figure 4B). Co‐immunofluorescence imaging for GFP and α‐SMA showed that COUP‐TFII had no clear influence on the SMC differentiation (Figure 4C). Based on the above findings, it was necessary to investigate the primary function of the cells that originated from BM. The results of co‐immunofluorescence staining for GFP and CD45 stated that approximately one‐third of the leukocytes present in the grafts originated from the BM (Figure 4D). Collectively, these data demonstrated that cells from BM partially contributed to vascular regeneration but mainly contributed to inflammatory responses after graft implantation, although overexpression of COUP‐TFII had a positive effect on promoting BM‐derived cells into endothelial lineage.

Co‐immunofluorescence staining for RFP and CD31 proved that a subset of the CD31^+^ cells (VP: 23.44% ± 4.85% vs CP: 39.85% ± 4.75%) were derived from resident Sca‐1^+^ cells (i.e., SPCs) and COUP‐TFII overexpression significantly promoted the endothelial differentiation of resident Sca‐1^+^ cells (Figure 4E). Co‐immunofluorescence staining for RFP and α‐SMA indicated that the majority of α‐SMA^+^ cells (VP: 60.37% ± 5.18% vs CP: 53.01% ± 4.89%) were derived from resident Sca‐1^+^ cells, but COUP‐TFII had no significant impact on the regeneration of the smooth muscle by resident Sca‐1^+^ cells (Figure 4F). These results suggested that resident SPCs were the primary contributors to vascular regeneration, including functional endothelium and an organized smooth muscle layer after graft implantation. Additionally, COUP‐TFII appeared to have a positive effect on endothelial differentiation without affecting the SMC differentiation potential of resident Sca‐1^+^ cells.

### 2.5 Fabrication and Characterization of Decellularized Allografts Loaded with COUP‐TFII@HPEI Nanoparticles

**Figure 5:**
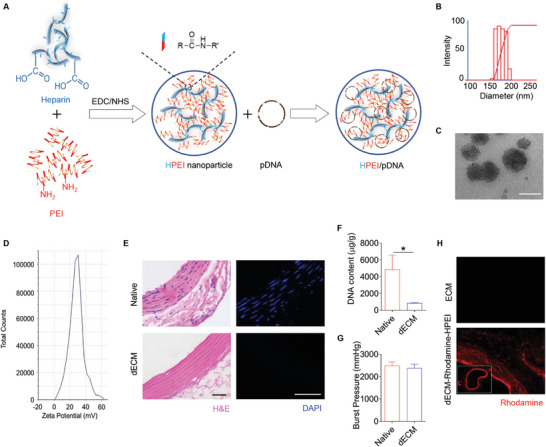
Fabrication and characterization of decellularized allografts functionalized with COUP‐TFII@HPEI nanoparticles. A) Schematic illustration shows the fabrication of heparin‐polyethyleneimine (HPEI) nanoparticles. B) Dynamic light scattering analysis of the HPEI nanoparticles is shown. C) Transmission electron microscope image shows the morphology of HPEI nanoparticles. Scale bar: 100 nm. D) Zeta potential of HPEI nanoparticles is shown. E) Shown are H&E and DAPI staining of the native and decellularized allografts, respectively. Scale bar: 50 µm. F) DNA quantification of native vessels and decellularized allografts is shown (*n* = 3). G) Burst pressure of the native and decellularized allografts is shown (*n* = 4). (H) Fluorescence images show the distribution of Rhodamine‐HPEI nanoparticles in the decellularized allograft. Scale bar: 50 µm. All data are presented as the means ± SEM. *p < 0.05.

To realize long‐term and stable delivery of COUP‐TFII in vivo, HPEI nanoparticles were further prepared via condensation of low molecular weight heparin and polyethyleneimine (PEI) using NHS/EDC as crosslinking agents (**Figure** **5**A). The chemical structure of as‐prepared HPEI nanoparticles was characterized by infrared spectroscopy (Figure S9A, Supporting Information). Dynamic light scattering (DLS) showed the hydrodynamic diameter of nanoparticles was 177 ± 11 nm (Figure 5B). Spherical morphologies of HPEI nanoparticles were further observed by transmission electron microscopy (TEM) with a diameter of 83.53 ± 16.09 nm in dry state (Figure 5C). The incorporation of heparin effectively reduced the cytotoxicity of low molecular weight PEI (*M_w_* 2000) owing to the reduced content of primary amine groups,^[^ ^44^, ^45^ ^]^ while retaining an overall positive charge (Figure 5D) to enhance effective uptake and transfection. Cell viability assays showed a pDNA:HPEI mass ratio‐dependent decrease in cell viability (Figure S9B, Supporting Information). To evaluate the binding ability of HPEI with pDNA, a gel retardation assay was performed. When the mass ratio of HPEI to pDNA exceeded 1, no DNA band was observed, suggesting that the DNA had been completely incorporated into HPEI (Figure S9C, Supporting Information). Next, we evaluated the transfection efficiency of HPEI nanoparticles using an eGFP overexpressing plasmid. Flow cytometric analysis demonstrated that the transfection efficiency of HPEI nanoparticles was highest when the mass ratio of pDNA:HPEI was 1:50 and 1:100 (Figure S9D, Supporting Information). Considering cytotoxicity, adsorption efficiency, and transfection efficiency, the optimal mass ratio of pDNA:HPEI was determined to be 1:50. HPEI nanoparticles were then loaded with pDNA encoding Myc‐tagged COUP‐TFII (Figure S10A, Supporting Information).

Rat abdominal aortas were harvested from donor rats and decellularized by a relatively mild yet effective method that can maintain the mechanical properties of vascular tissues sufficiently even after the decellularization treatment (Figure S10B, Supporting Information). H&E and DAPI staining demonstrated that nuclei were successfully eliminated, while indicated that the ECM (extracellular matrix) microstructure was well preserved which has been further confirmed by SEM imaging (Figure 5E; Figure S10C, Supporting Information). The efficiency of decellularization was further confirmed through DNA quantification (Figure 5F). Burst pressure and tensile strength measurements verified that the decellularized vessels retained sufficient mechanical strength for application as arterial replacements (Figure 5G; Figure S10D, Supporting Information). Successful binding COUP‐TFII@HPEI nanoparticles within the decellularized allografts was also demonstrated by fluorescence detection using Rhodamine‐labeled HPEI nanoparticles (Figure 5H). In vitro release assay exhibited a rapid release of ≈60% COUP‐TFII@HPEI nanoparticles from decellularized allografts during the initial period of 10 h with the residency remained within the allografts over the course of 7 days due to the stable and effective adsorption (Figure S10E, Supporting Information).

### 2.6 COUP‐TFII Increases Endothelialization of Decellularized Allografts in Rats

**Figure 6:**
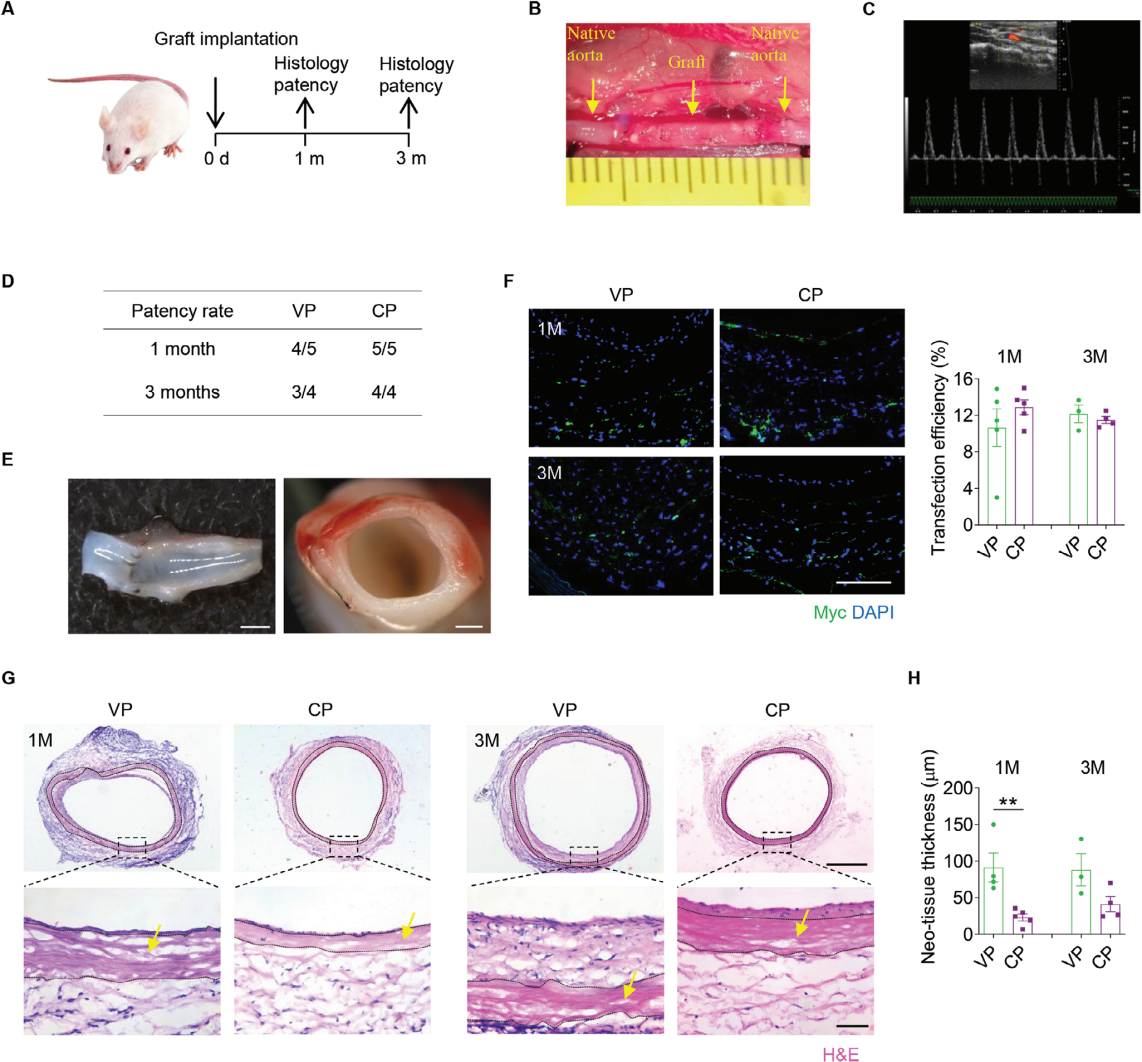
In vivo evaluation of decellularized allografts functionalized with COUP‐TFII@HPEI nanoparticles in a rat abdominal artery replacement model. A) Experimental schedule for implantation and evaluation in a rat abdominal artery replacement model. B) Representative image shows the graft after implantation. C) Ultrasound image of the implanted graft is shown. D) Statistic analysis of the patency rates of implanted vascular grafts. E) Stereoscopic images show the grafts explanted at 3 months. Scale bar: 1 mm (Left) or 500 µm (Right). F) Shown is immunofluorescence staining for Myc tag with quantitative analysis of the transfection efficiency (*n* = 3–5). Scale bar: 100 µm. G) H&E staining of the explanted grafts is shown. Yellow arrows show the implanted allografts. Scale bar: 500 or 50 µm (magnified images). H) The thickness of neo‐tissue was quantified (*n* = 3–5). All data are presented as the means ± SEM. **p < 0.01.

The regenerative performance of the decellularized allografts functionalized with COUP‐TFII@HPEI nanoparticles was evaluated in a rat abdominal artery replacement model (**Figure** **6**A,B). The patency of the implanted grafts was evaluated by Doppler ultrasound (Figure 6C). Both VP and CP groups maintained high graft patency rates after 1 and 3 months of implantation, with CP grafts exhibiting a slightly higher patency than the VP grafts (Figure 6D). The explanted vascular grafts were first observed by stereoscopic microscopy. Images showed that the grafts had smooth lumens without thrombosis (Figure 6E). Myc‐tagged COUP‐TFII was visualized by immunofluorescence staining, which indicated that the transfection efficiency of COUP‐TFII@HPEI was similar (≈10%) in the groups at two different time points (Figure 6F). Then, H&E staining illustrated that COUP‐TFII significantly (*p* < 0.01) attenuated intimal hyperplasia after 1 month of implantation, although the difference between the two groups narrowed over time (Figure 6G,H).

**Figure 7:**
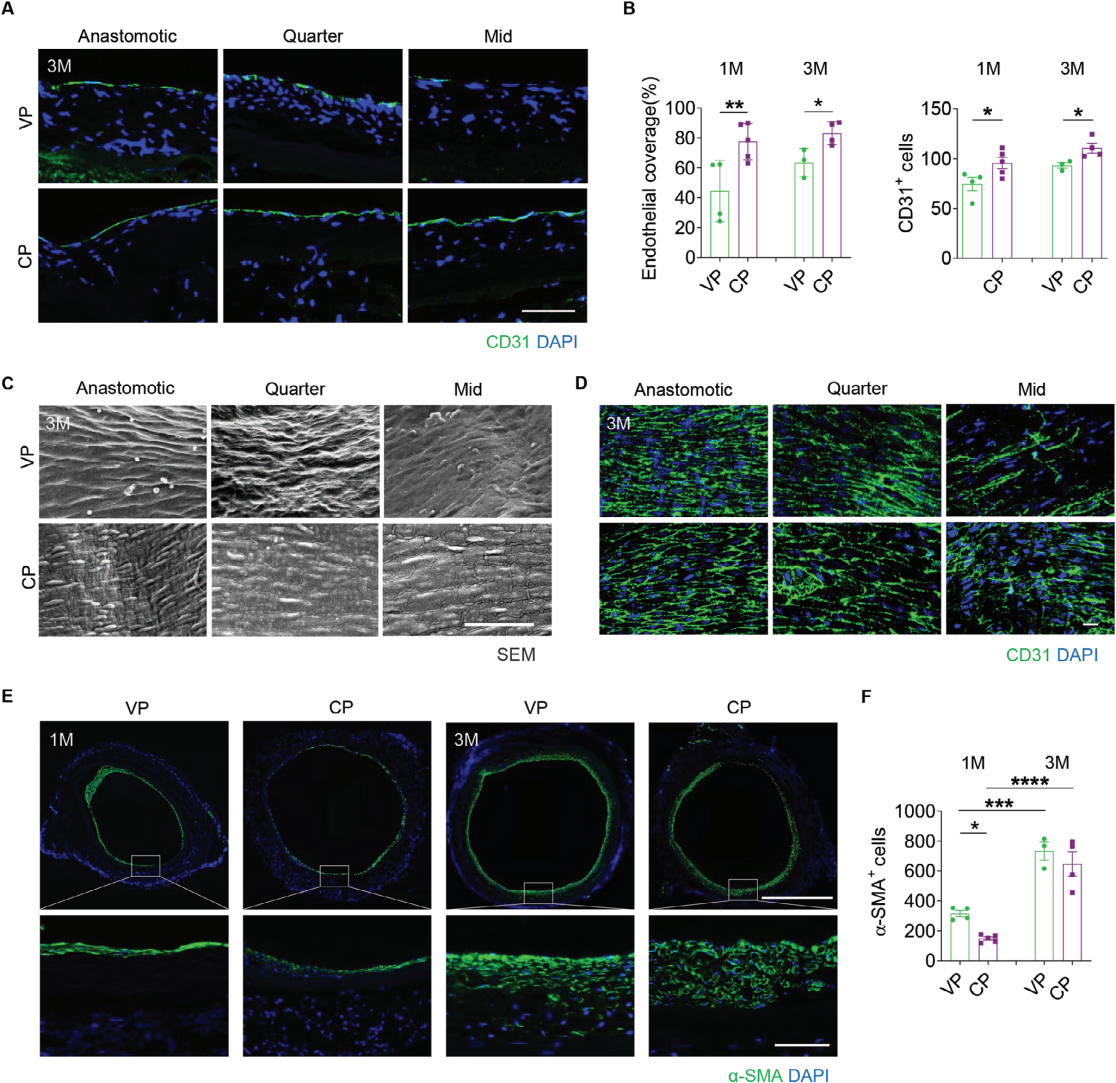
COUP‐TFII enhances endothelialization of decellularized allografts. A) Immunofluorescence staining for CD31 is shown. Scale bar: 50 µm. B) The endothelial coverage ratio and the number of CD31^+^ cells were quantified (*n* = 3–5). C) SEM images show the spindle ECs lining on the lumen of grafts. Scale bars: 50 µm. D) En‐face immunofluorescence staining for CD31 is shown. Scale bar: 20 µm. E) Immunofluorescence staining for α‐SMA of the explanted grafts is shown. Scale bars: 1 mm or 50 µm (magnified images). F) The number of α‐SMA^+^ cells was quantified (*n* = 3–5). All data are presented as the means ± SEM. *p < 0.05, **p < 0.01, ***p < 0.001, ****p < 0.0001. Anastomotic, Quarter, and Mid represent the anastomotic, quarter, and midportion sites of the explanted vascular grafts.

The endothelialization of the allografts was evaluated by immunofluorescence staining for CD31 (**Figure** **7**A; Figure S11, Supporting Information). Quantitative analyses of the number of CD31^+^ cells and the ratio of the length covered by CD31^+^ endothelial cells to the total length of the allograft surface indicated that COUP‐TFII accelerated endothelialization of the decellularized allografts (Figure 7B). The SEM images showed that the luminal surface of grafts functionalized with COUP‐TFII@HPEI (CP) had a higher degree of coverage by cobblestone‐shaped cells, compared to the control group (VP) (Figure 7C). The formation of intercellular junctions by ECs was evident in the CP grafts, and this contrasted with VP grafts, as validated by en face immunofluorescence staining for CD31 (Figure 7D). The regeneration of a smooth muscle was evaluated by immunofluorescence staining for α‐SMA, which supported the COUP‐TFII slowed the regeneration of smooth muscle in the initial stage after implantation, which may restrict the VSMC overgrowth that leads to intimal hyperplasia, while in the later stage, the smooth muscle layers were also well organized as the control group (Figure 7E,F).

### 2.7 COUP‐TFII Effectively Attenuates Inflammation and Calcification of Decellularized Allografts in Rats

**Figure 8:**
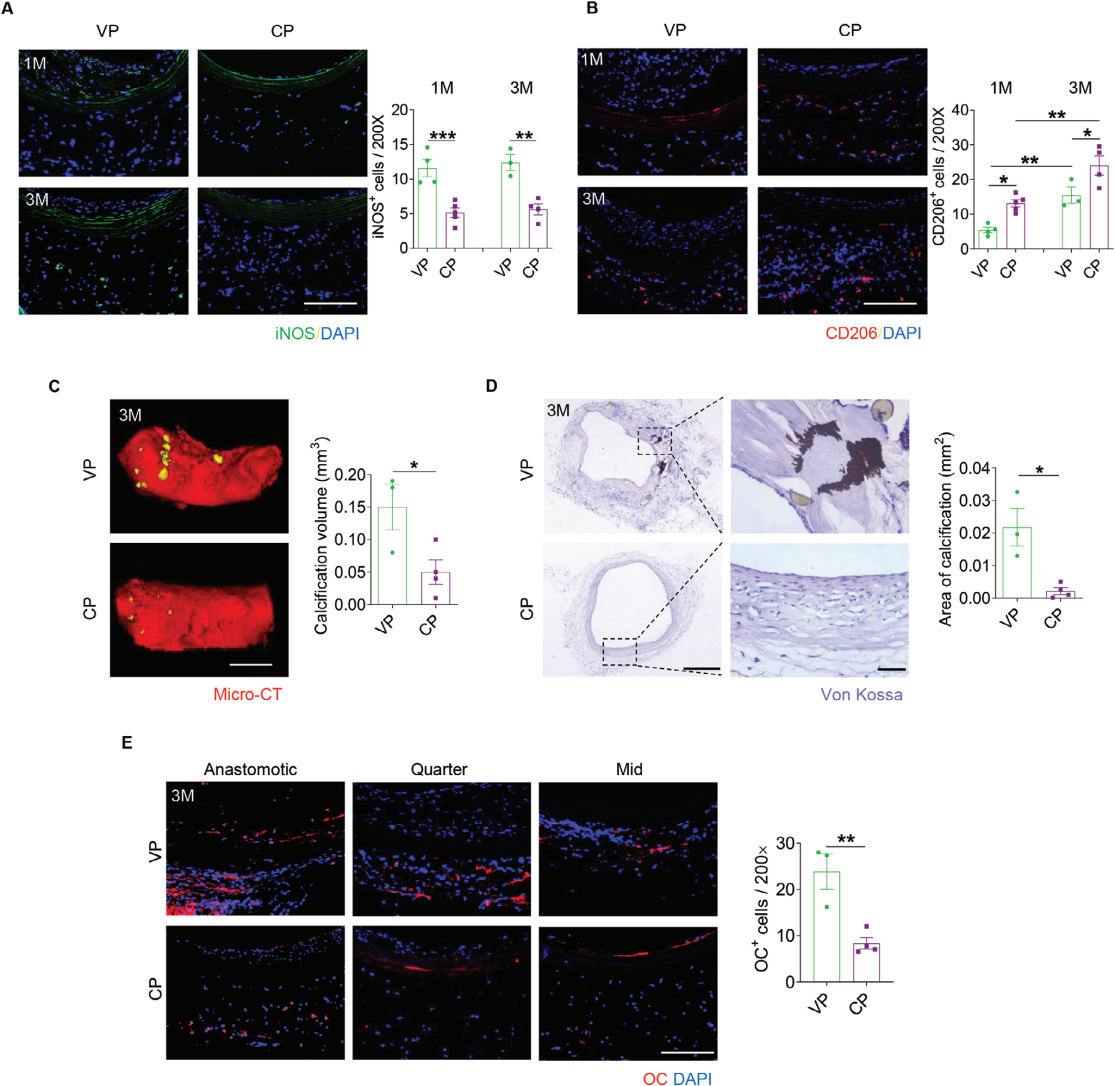
COUP‐TFII attenuates inflammation and calcification of decellularized allografts. A) Shown is immunofluorescence staining for iNOS and quantification of the number of iNOS^+^ cells (*n* = 3–5). Scale bar: 100 µm. B) Immunofluorescence staining for CD206 is shown. And the number of CD206^+^ cells was quantified (*n* = 3–5). Scale bar: 100 µm. C) Micro‐CT images of grafts explanted at 3 months are shown and the calcification volume was quantified (*n* = 3 or 4). Scale bars: 1 mm. D) Shown is Von Kossa staining images of grafts explanted at 3 months and quantification of the Von Kossa positive area (*n* = 3 or 4). Scale bars: 500 or 50 µm (magnified images). E) Immunofluorescence staining for OC of the graft wall is shown. And the number of OC^+^ cells was quantified (*n* = 3 or 4). Scale bar: 100 µm. All data are presented as the means ± SEM. *p < 0.05, **p < 0.01, ***p < 0.001.

To explore the inflammatory response to decellularized allograft after implantation, immunofluorescence staining for iNOS and CD206 was performed to detect the distribution of M1 and M2 macrophages in the different groups at two‐time points. The results indicated that COUP‐TFII increased the infiltration of M2 macrophages, while reducing the number of M1 macrophages present in the allografts, suggesting a modulated inflammatory response in favor of vascular regeneration (**Figure** **8**A,B). The calcification within the graft wall was first assessed by micro‐CT. The quantification of the calcification volume reflected a pronounced inhibition of COUP‐TFII on the calcification (Figure 8C). Further histological analyses, including Von Kossa staining and immunofluorescence staining for OC supported the inhibitory effect of COUP‐TFII on ectopic calcification of decellularized allografts (Figure 8D,E).

## 3 Discussion

Successful endothelialization of vascular grafts facilitates to mimic the luminal structure and functions of natural blood vessels, which in turn, is conducive to providing long‐term patency of the grafts while simultaneously reducing the risk of adverse remodeling.^[^ ^46^, ^47^ ^]^ However, vascular grafts made from synthetic or natural polymers often lack the growth factors and signaling cues present in natural vessels, which are crucial for vascular endothelial cells proliferation, migration, and differentiation,^[^ ^48^, ^49^ ^]^ thereby impeding the endothelialization progress after implantation. Thus, strategies to endow vascular grafts with enhanced capabilities to achieve endothelialization can help to provide improved outcomes and reliability for their clinical application.^[^ ^7^, ^12^, ^24^, ^50^, ^51^ ^]^

Arteries and veins play distinct roles in human physiology, and arterial and venous ECs have unique molecular profiles that maintain their specific physiological functions in their own environment.^[^ ^40^ ^]^ Venous ECs demonstrate superior antithrombotic actions and express fewer proinflammatory and osteogenic factors, which may protect venous endothelium against vascular lesion such as atherosclerosis and vascular calcification.^[^ ^41^, ^52^ ^]^ Previously, arterial and venous ECs were thought to derive from common endothelial progenitor cells (angioblasts), and that arterial or venous identities were acquired during development.^[^ ^53^, ^54^ ^]^ Multiple factors have been identified to play pivotal roles in arteriovenous differentiation, such as the Notch and BMP signaling pathways,^[^ ^30^, ^38^, ^39^, ^55^ ^]^ EC cycle status,^[^ ^56^, ^57^ ^]^ and others. However, recent studies have demonstrated that arterialization from the capillaries with considerable venous characteristics, providing new insights into studying adult arteriogenesis.^[^ ^58^ ^]^ In the present study, we sought to endow the newly formed endothelium with venous characteristics to accelerate the comprehensive regeneration of vascular tissues and to protect against the development of adverse vascular remodeling commonly faced by implanted graft materials.

The venous transcription factor, COUP‐TFII (also known as NR2F2), regulates the fate of arterial‐venous differentiation and pathophysiological functions. In the absence of COUP‐TFII, arterial markers appear ectopically in the venous circulatory system. Meanwhile, COUP‐TFII inhibition of Notch signaling was shown to play a role in arterial specification of ECs.^[^ ^38^, ^39^, ^40^, ^41^ ^]^ Furthermore, COUP‐TFII acts as a suppressor of pro‐atherogenic and osteogenic potential in vessels and thus may play a key role in the differential susceptibility of arteries and veins to vascular diseases such as atherosclerosis and vascular calcification.^[^ ^43^, ^59^ ^]^ In human primary endothelial cells, NR2F2 suppresses DKK1, while its loss induces DKK1 and disrupts endothelial homeostasis, promoting the phenotypic abnormalities associated with pathological vascular remodeling. Meanwhile, after influenza injury, nuclear factor κB inhibits COUP‐TFII, but surviving ECs ultimately rely on recovery of COUP‐TFII to reestablish vascular homeostasis.^[^ ^60^ ^]^ These previous studies supported the implementation of COUP‐TFII over‐expression as a strategy to promote endothelialization and mitigate risk of adverse remodeling occurrence in our developed decellularized allografts.

In this study, we first isolated a type of Sca‐1^+^ SPCs from the adventitial of the rat aorta and demonstrated that COUP‐TFII promotes the differentiation of Sca‐1^+^ SPCs into ECs in vitro via activation of the Ang1/Tie2/PI3K/AKT axis. The pDNA encoding COUP‐TFII was further loaded into decellularized allografts using different carriers and effect of overexpressed COUP‐TFII on the vascular regeneration and remodeling has been comprehensively investigated in both mouse models of right common carotid artery replacement and rat models of abdominal aorta replacement. We demonstrated that over‐expression of COUP‐TFII effectively promoted endothelialization, whereas inhibited intimal hyperplasia and vascular calcification of decellularized allografts via regulating the differentiation of endogenous Sca‐1^+^ SPCs.

To get further insight into the origin and fate of the Sca‐1^+^ SPCs participating in the regeneration and remodeling of the implanted grafts, we established a reconstituted BM chimera mouse model in which BM from CAG‐EGFP mice were transplanted into *Sca‐1 2A‐CreER; Rosa‐RFP* lineage‐tracing mice. In vivo investigation demonstrated that resident SPCs were the primary contributors to vascular regeneration via differentiation into ECs and SMCs in the grafts. In contrast, cells from BM mainly contributed to inflammatory responses, while partially involving in vascular regeneration, including endothelium and vascular smooth muscle, after graft implantation. Furthermore, locally overexpressed COUP‐TFII demonstrated a modulatory effect on the endothelial differentiation of both BM and non‐BM‐derived cells.

In summary, these findings offer a novel and simple approach to fabricating functionalized acellular vascular grafts that may serve as easily translational and promising candidates for pro‐regenerative small‐diameter vascular grafts. Moreover, our studies validated that delivery of the venous endothelial transcription factor COUP‐TFII offers an effective strategy for the vascular regeneration, wherein neo‐tissue formation and homeostasis maintenance are requisite. Inspired by the distinct functions of the Sca‐1^+^ cells derived from different origins, in‐depth future studies on the precise control of multi‐lineage commitment are needed to further realize the potential implications for mobilizing endogenous Sca‐1^+^ SPCs in the treatment of vascular diseases.

## 4 Experimental Section

### Construction of Plasmid Vectors

The genes encoding COUP‐TFII (NR2F2) derived from mouse and rat (NM_080778.2 and NM_009697.3) were codon‐optimized (for the *Escherichia coli* expression system) and synthesized by Origene (USA) to obtain COUP‐TFII pDNA (CP) and Vacant pDNA (VP). Competent *Escherichia coli* were transformed with the plasmids and amplified in LB liquid medium containing corresponding antibiotics and cultured at 220 rpm at 37 °C for 12 h. The cloned plasmids were extracted using a plasmid extraction kit (TIANGEN, China), according to the product instructions.

### Fabrication of HPEI Nanoparticles

The preparation method of HPEI nanoparticles referred to existing literature.^[^ ^44^, ^61^ ^]^  Low molecular weight heparin (*M_w_* = 4500, 50 mg) (ALFA WASSERMANN, Italy) was first dissolved in 0.05 mol L^−1^ MES (pH5.5, 100 mL), in which 10 mg EDC and 30 mg NHS were added, and the solution was placed in ice bath in a round‐bottom flask and stirred for 1.5 h to activate the carboxyl groups on heparin sodium. Then a volume of 20 mL 7.5 mg mL^−1^ PEI2000 solution (Sigma–Aldrich, USA) was added to the above solution slowly and uniformly, then 10 mg EDC was added. The process was performed under continuous stirring and on ice, to avoid aggregation. Following continuous stirring on ice overnight, the solution was transferred to a dialysis bag (MWCO = 8000–14 000) and dialysis was performed with distilled water at RT for 3 days, with daily water changes. The dialyzed solution was filtered by a filter membrane with a pore size of 450 nm, and the concentration of the nanoparticle solution was adjusted to 1 mg mL^−1^ before storage at 4 °C until bone marrow transplantation.

### Characterization of HPEI Nanoparticles

Lyophilized low‐molecular‐weight heparin, PEI, and HPEI nanoparticles were analyzed by Fourier transform infrared spectroscopy (TENSOR II, Bruker, Germany). The particle size and zeta potential of HPEI nanoparticles in solution were detected by Dynamic light scattering and Electrophoretic light scattering (ZETAPALS/BI‐200SM, BROOKHAVEN, USA). For transmission electron microscopy (HT7700 Exalens, Hitachi, Japan) visualization, 20 µL of the solution of HPEI nanoparticles was dropped onto copper wire and allowed to dry at RT before TEM detection.

The ability of the HPEI nanoparticles absorb plasmid DNA of different indicated mass ratios of HPEI:pDNA was evaluated by Electrophoretic Mobility Shift Assay (EMSA).

The cytotoxicity of different indicated mass ratios of pDNA: HPEI was evaluated by Cell Counting Kit‐8 assay in 3T3 cells. 3T3 cells were seeded at a density of 1000 cells per well in 100 µL of media into 96‐well plates, and the transfection procedure was as described above. The cytotoxicity of the HPEI nanoparticles was evaluated by Cell Counting Kit‐8 assay (Beyotime, China). In detail, the medium of each well was removed and replaced with 100 µL of CCK8 dilution containing 10 µL of CCK8 and 90 µL of DMED complete medium, then maintained at 37 °C for 4 h. Finally, the optical density value (OD450) was measured using a microplate reader (Synergy 4, BioTek, USA).

The transfection efficiency of different indicated mass ratios of pDNA:HPEI was evaluated by flow cytometry in 293T cells. Fresh 293T cell culture medium was formulated with 5 mL FBS (Gibco, USA), 500 µL penicillin/streptomycin solution (Gibco, USA) and 1 mL L‐glutamine solution (Gibco, USA), and the volume was fixed to 50 mL with DMEM high glucose medium (Gibco, USA). The 293T cells were seeded on six‐well plates at a density of 2 × 10^5^ cells per well and incubated in culture medium for 24 h. The eGFP labeled CP was transfected into 80–90% confluent 293T cells using HPEI nanoparticles with the different indicated mass ratios of pDNA:HPEI. After transfection in serum‐free medium for 6 h, culture was replaced with normal medium. After an additional 48–72 h, the cells were collected for subsequent analyses. The collected cells were washed with PBS and counted. Then, cells were fixed with 4% paraformaldehyde at RT for 15 min. After two washes with PBS and filtration with a 200‐mesh filter, the transfection efficiency of eGFP labeled CP was determined by flow cytometry.

### Fabrication of Decellularized Blood Vessels

Great abdominal aortas were freshly harvested from healthy and age‐matched mice and rats, respectively. The branches of the mouse thoracic aorta were blocked by electrocoagulation, while for the rat abdominal aorta, the branches were ligated by 9‐0 monofilament nylon sutures. Decellularization was performed by treating the mouse and rat vessels with 0.04% or 0.1% SDS (Sangon Biotech, China) in a shaker for 4 h at RT, respectively. Subsequently, they were washed thoroughly with saline at RT. The mouse and rat vessels were soaked in 0.05 or 0.1 mg mL^−1^ DNase solution in a shaker for 2 or 12 h at RT, respectively. They were washed thoroughly with saline at RT once again. The decellularized vessels were further modified by heparinization in PBS solution containing 1 mg mL^−1^ heparin, 5.751 mg mL^−1^ EDC, and 5.179 mg mL^−1^ NHS at 4 °C for 10 h, followed by washing with saline three times at 4 °C. The heparinized rat vessels were subsequently treated by vacuum freeze‐drying, immersion in a solution containing COUP‐TFII@HPEI for 12 h, followed by storing in 1% penicillin‐streptomycin PBS solution at 4 °C overnight.

### Characterization of Decellularized Blood Vessels

For DNA residue quantification, three sections were taken from dried blood vessels before and after decellularization. The initial mass of each sample was weighed and recorded (marked as M). Then, each section of blood vessel was cut with ophthalmic scissors and placed in a 1.5 mL microcentrifuge tube. According to the extraction procedure of the tissue DNA extraction kit, the total DNA of each sample was extracted and dissolved in ddH_2_O of a certain volume (marked as V). Subsequently, DNA concentration (marked as C) of each sample was determined using a Nano Drop device (Nanodrop 2000, ThermoFisher Scientific, USA). Then, the DNA content (η) was calculated using η = VC/M. The mechanical properties of the grafts were measured on a tensile‐testing machine (Instron, USA). Grafts with 1 cm in length were fixed on two steel rings which were clamped and pulled longitudinally at a rate of 10 mm s^−1^ until rupture. Subsequently, four sections of blood vessels before and after decellularization were used to detect burst pressure. One end of the vessel was ligated with 3‐0 surgical suture and the other end was fixed with a flattened needle of a 20 mL syringe. The vascular cavity was coated with Vaseline sealant and the pinning needle was then removed. Then the vessel was connected to a nitrogen cylinder via a size‐matched pipe. The blood vessel cavity was filled by nitrogen at the rate of 0.1 mL min^−1^. The pressure sensor recorded the air pressure inside the blood vessel in real time until the blood vessel burst.

### Rat Sca‐1^+^ SPCs Isolation

Rat SPCs were isolated from aortic adventitial tissue as previously described.^[^ ^62^ ^]^ Briefly, the aortic arch and root from SD rats were harvested under sterile conditions. The adventitia was carefully detached from the media and intima layers, cut into pieces and seeded onto gelatin‐coated flask, and cultured with DMEM (ATCC, USA) containing 20% ES Cell Qualified Fetal Bovine Serum (FBS) (Gibco, USA), 10 ng mL^−1^ LIF (Merck Millipore, USA), 0.1 mm β‐mercaptoethanol (Merck Sigma–Aldrich, USA), and 100 U mL^−1^ penicillin/streptomycin (Gibco, USA). After reaching 90% confluency, cells were selected for the Sca‐1^+^ marker using anti‐Sca‐1 immunomagnetic microbeads (Miltenyi Biotec, Germany), sorted with a magnetic cell separator following the manufacturer's instructions. Cells were sorted for Sca‐1^+^ marker every five passages.

### Transfection and Differentiation of Sca‐1^+^ SPCs

COUP‐TF II–specific siRNA (GenePharma, 5′‐CCACAUACGGAUCUUCCAATT‐3′ and 5′‐UUGGAAGAUCCGUAUGUGGTT‐3′) and overexpression plasmids were generated and transfected as previously reported. SPCs were plated in stem cell media for 3 days. At day 4, SPCs were transfected with COUP‐TF II–specific siRNA or plasmids using the Lipofectamine 2000 transfection kit (Biosharp, China), according to the manufacturer's instructions. Then, the medium was replaced with a differentiation medium composed of DMEM supplemented with 100 U mL^−1^ penicillin/streptomycin, 10% FBS, and added 5 µm Tie2 inhibitor (GLPBIO, USA) or 20 µm PI3K inhibitor (MCE, USA) in the subsequent experiments. At 10 h or 4 days later, cells were harvested for RT‐qPCR and Western blot or fixed for immunofluorescence staining.

### RNA Extraction and RT‐qPCR

Total RNA was harvested according to the MolPure Cell RNA Kit (YEASEN, China) protocols, and the total RNA solution was reverse transcribed to cDNA using the Hifair III 1st Strand cDNA Synthesis SuperMix (with gDNA digestion) kit (YEASEN, China) following the manufacturer's protocols. The cDNA was stored at −20 °C until analysis by RT‐qPCR using a CFX96TM Real‐Time PCR System (LightCycler 96, Roche, Switzerland) with the SYBR Green‐based real‐time detection kit (YEASEN, China). The relative expression level of the mRNA of interest was expressed as 2^−(ΔΔCt)^ and GAPDH was used as an endogenous control. The primer sequences used in this study are listed in Table S1 (Supporting Information).

### Protein Extraction and Western Blot

SPCs were lysed using RIPA buffer supplemented with PMSF and protein phosphatase inhibitors (Solarbio, China). The lysate was transferred to microcentrifuge tubes and incubated on ice for 30 min with oscillation every 5 min. Next, the samples were centrifuged at 12000 r min^−1^ for 15 min and then the supernatant was transferred to a new 1.5 mL tube. The protein concentration was quantified by a BCA Kit (Solarbio, China). Equal amounts of total protein were electrophoresed with 10% SDS‒PAGE and transferred onto PVDF membranes (Merck Millipore, USA). The membranes were blocked with 5% nonfat milk for 45 min and incubated with the corresponding primary antibodies at 4 °C overnight. The following primary antibodies were used in this work: mouse anti‐β‐actin (ZSGB‐Bio, TA346894), mouse anti‐α‐SMA (Abcam, ab7817), rabbit anti‐CNN1 (Abclonal, A3734), rabbit anti‐CD31 (Abcam, ab222783), rabbit anti‐Tie2 (Abclonal, A7222), rabbit anti‐Ang1 (Abclonal, A15026), rabbit anti‐AKT (Cell Signaling, 4060), rabbit anti‐p‐AKT (Cell Signaling, 4691). After rewarming for 1 h, PBST was used to wash the membrane six times, followed by incubation with HRP‐conjugated secondary antibodies (ZSGB‐Bio, ZB‐2305, and ZB‐2301) at room temperature for 2 h. Immunoreactive bands were visualized with an ECL immunoblotting kit (Merck Millipore, USA). ImageJ software was used to quantify the protein expression level.

### Immunofluorescent Staining of Differentiated Sca‐1^+^ SPCs

The SPCs were planted into 48‐well plate at a density of 2.5 × 10^4^ cells per well, followed by transfection and cultured as described above. After 4 days, the cells were washed with PBS and fixed with 4% paraformaldehyde. Then, the cells were stained using polyclonal anti‐CD31 (Abcam, ab64543) or anti‐calponin (Abcam, ab46794) primary antibodies followed by Alexa Fluor 488‐conjugated goat anti‐mouse IgG (Invitrogen, A‐11001) or Alexa Fluor 594‐conjugated goat anti‐rabbit IgG (Invitrogen, A‐11012), respectively.

### Rats and Mice

Wild‐type C57BL/6 mice (WT mice, male, 20–25 g) and Sprague–Dawley rats (SD rats, male, 280–320 g) were obtained from Beijing Sipeifu Biotechnology Co., Ltd. Transgenic mice with systemic EGFP expression (CAG‐EGFP mice, male, 20–25 g) were purchased from Shanghai Model Organisms Center, Inc. *Sca‐1 2A‐CreER* mice (C57BL/6 background, female and male, 20–25 g) and *Rosa‐RFP* mice (C57BL/6 background, female and male, 20–25 g) were kindly provided by Professor Bin Zhou from Shanghai Institute of Biochemistry and Cell Biology.^[^ ^63^, ^64^ ^]^ The use of experimental animals was approved by the Animal Experiments Ethical Committee of Nankai University and conducted according to the Guide for Care and Use of Laboratory Animals.

### Mice Breeding and Genotyping

*Sca‐1 2A‐CreER; Rosa‐RFP* lineage tracing mice were obtained by crossing *Sca‐1 2A‐CreER* mice with *Rosa‐RFP* mice. All male and female mice were used at 8 weeks old in this study. Toe samples of the offspring of *Sca‐1 2A‐CreER* mice and *Rosa‐RFP* mice were collected for total genomic DNA extraction using the TIANamp Genomic DNA Kit (TianGen, China). Polymerase chain reaction was performed to amplify target genes using 2×Taq PCR Colorless Mix (CWBIO, CW0690 M). The primers sequences of *Sca‐1 2A‐CreER* were pr4857: 5′aacgtgaagacttcctgttg‐3′, pr4858: 5′‐cacacactactcccaccttg‐3′ and pr4859: 5′‐ggtttccctgccacagcttg‐3′. The primers sequences of *Rosa‐RFP* were pr1590: 5′‐aagggagctgcagtggagta‐3′, pr1591: 5′‐ccgaaaatctgtgggaagtc‐3′, pr1592: 5′‐ggcattaaagcagcgtatcc‐3′ and pr1593: 5′‐ctgttcctgtacggcatgg‐3′. Then, the amplification products were analyzed by agarose gel electrophoresis. The gene size of *Sca‐1 2A‐CreER* was 1049 bp compared to wild type 246bp. The gene size of *Rosa‐RFP* was 196 bp compared to wild type 297 bp. Mice with positive expression of both genes were identified as *Sca‐1 2A‐CreER; Rosa‐RFP* lineage tracing progenies.

### Bone Marrow Cell Isolation

Mouse femurs and humerus bones were sterilized in 75% alcohol, transferred to petri dish, and immersed in sterile PBS. The procedure was performed on an ultra‐clean workbench. Then, the muscle on the surface of the bone tissue was completely removed before replacing the petri dish and PBS. The bone marrow was then exposed by cutting both ends of the femur and humerus, and PBS was injected with a syringe from one end of the bone to flush out and collect the internal marrow cells from the other end. The collected cells were pelleted, re‐suspended in sterile PBS, and adjusted to a cell concentration of 2 × 10^7^/mL after removing red blood cells by lysis and centrifugation. The final cell suspension was placed on ice before bone marrow transplantation.

### Bone Marrow Transplantation

Two weeks after tamoxifen induction, *Sca‐1 2A‐CreER; Rosa‐RFP* mice were irradiated with gamma rays to destroy cells. The dose was 4 Gry twice at an interval of 4 h. At 6 h after irradiation, freshly extracted GFP^+^ mouse BM cells were injected into the tail vein of the mice exposed to radiation. Each mouse was injected with 5 × 10^6^ cells (approximately volume, 250 µL). Mice receiving BM transplantation were kept in clean sterile cages and bedding. Enrofloxacin was fed in drinking water.

### Bone Marrow Reconstruction Rate Evaluation

After 4 weeks and 6 weeks, 40 µL blood was taken from tail and dissolved in 80 µL 2% EDTA solution for anticoagulation. After the removal of red blood cells, the percentage of GFP^+^ cells in the blood of the mice was measured by flow cytometry, which showed the BM reconstruction rate. As a general rule, only mice with reconstruction efficiency above 90% were used as reconstituted BM chimera mice in carotid artery replacement models.

### Loading of Plasmid DNA in Decellularized Allografts

For the decellularized allografts used in the mouse carotid artery replacement model and the reconstituted bone marrow chimera mouse carotid artery replacement model, 50 mg of methyl acrylamide anhydride gelatin was dissolved in 500 µL initiator, at 58 °C for 30 min, with agitation every 5 min until the colloid components were completely dissolved.  A quantity of 4 µg pDNA was mixed with 50 µL PBS, and let stand for 5 min. Then, a volume of 10 µL Lipofectamine 2000 was added to 50 µL PBS solution, mixed gently, and let stand for 5 min.  The DNA solution was then added to the Lipofectamine 2000 solution, mixed, and then allowed to stand for 20 min. Finally, the prepared 10% liquid gelatin methacryloyl (GelMA) (Engineering for Life, China) was fully mixed with DNA/Lipofectamine 2000 transfection complex. After carotid artery implantation in mice, 50 µL of the transfection complex was added to the outer wall of the decellularized allografts, and the mixture was immediately illuminated with ultraviolet light for 20 s to crosslink the gel on the outer membrane of the decellularized allografts, realizing the loading of pDNA on the decellularized allografts.

For the decellularized allografts used in the rat abdominal artery replacement model, the positively charged HPEI nanoparticles and the negatively charged pDNA were mixed evenly according to the 50:1 mass ratio and stood for 15 min. Under the principle of electrostatic interaction, the two components form COUP‐TFII@HPEI nanoparticles. Then, the prepared decellularized blood vessels of rat abdominal aorta were vacuum freeze‐dried before immersion in a solution containing COUP‐TFII@HPEI for 12 h, yielding DNA/HPEI loaded grafts. In vitro release assay of COUP‐TFII@HPEI from the decellularized grafts was exhibited using the Rhodamine‐HPEI in saline, at 37 °C.

### Implantation of Decellularized Allografts in a Mouse Model

The procedure used for grafts implantation was similar to that described previously.^[^ ^65^ ^]^ Mice were fasted for 24 h prior to surgery and were anesthetized using a combination of Hypnorm (25 mg kg^−1^; Veta Pharma, UK) and Hypnovel (25 mg kg^−1^; Roche, Switzerland) administered intraperitoneally. The mice were placed under the operating microscope in a supine position with limbs and head affixed to the surgical table and neck was extended. A midline incision was made extending from the clavicle to the chin and the salivary glands were separated to expose the trachea and right common carotid. The right common carotid artery was mobilized free from the bifurcation at the distal end toward the proximal, clamped at both ends, and cut in the middle, and a cuff placed over both ends. The cuff was made from an autoclavable nylon tube 0.63 mm in diameter outside and 0.5 mm inside (Portex Ltd., Hythe‐Kent, UK). The artery was turned inside out over the cuff and ligated. The decellularized allografts were grafted between the two ends of the carotid artery by sleeving the ends of the graft over the artery cuff and ligating them together with an 8‐0 suture. Finally, the blood flow was restored, and the muscle and skin incision were closed with 6‐0 nylon sutures. Aspirin was administrated daily as anti‐coagulant for 1 week (2 mg kg^−1^). The mice were kept in cages with food and water normally. The patency of the implanted vascular grafts was assessed at the indicated timepoints using high‐resolution ultrasound (Vevo 2100, Visual Sonics, Toronto, Canada), following rat anesthetization with isoflurane gas.

### Implantation of Decellularized Allografts in a Rat Model

The rats were fasted for 24 h prior to surgery. Rats were anesthetized by intraperitoneal injection of chloral hydrate (300 mg kg^−1^) before being placed under the operating microscope in a supine position, and limbs were affixed to the surgical table. Heparin (100 U kg^−1^) was administered by tail vein injection before surgery. A midline laparotomy incision (4–5 cm) was made and the visceral organs were separated to expose the abdominal aorta and left renal artery. The distal abdominal aorta between renal and common iliac artery was isolated, clamped at both ends, and transected in the middle. The grafts were anastomosed to the abdominal aorta and secured with an end‐to‐end fashion with 8–10 interrupted stitches using 9‐0 monofilament nylon sutures (Ling Qiao, Ningbo, China). Finally, blood flow was restored, visceral organs were restored, and the abdominal muscle and skin openings were surgically closed with 3‐0 nylon sutures. Rats were then placed into clean and dry cages on heating pads to maintain body temperature until the rats recovered from anesthesia. The patency of the implanted vascular grafts was assessed at the indicated timepoints using high‐resolution ultrasound (Vevo 2100, Visual Sonics, Toronto, Canada), following rat anesthetization with isoflurane gas.

### Histological Analysis

At predetermined timepoints, animals (mice or rats) were euthanized with over‐dose anesthesia and grafts were harvested. The implanted grafts, together with adjacent native aorta, were extracted and rinsed with saline to remove excess blood before observation by stereoscopic microscopy (Leica S8AP0, Wetzlar, Germany), and processed for histochemical analysis, as described in previous literature.^[^ ^66^ ^]^

For sample treatments, explanted grafts from rats were bisected. The whole explanted grafts from mice and one‐half of the explanted grafts from rats were fixed in 4% paraformaldehyde solution for 4 h. Then, graft samples were dehydrated in 30% sucrose solution for 12 h. Finally, they were embedded in OCT compound, frozen at −20 °C, and cryo‐sectioned into 6 µm thick sections (CM 1950, Leica, Germany). The other part of the explanted grafts from rats was longitudinally cut into two pieces. One piece was snap‐frozen in OCT for longitudinal section. The other piece was processed for SEM observation or en face immunofluorescence staining.

For SEM observation, the sample was fixed with 2.5% glutaraldehyde for 24 h, and dehydrated in a sequence of ethanol solutions, 5 min for each step. After air‐drying at RT, samples were sputter‐coated with gold for SEM analysis.

For en‐face immunofluorescence staining, the samples were fixed with 4% paraformaldehyde solution for 30 min, then incubated with 5% normal goat serum (Life Technologies, 01‐6201) for another 30 min at RT. Next, the samples were incubated with polyclonal anti‐CD31 primary antibody (Abcam, ab64543) in PBS overnight at 4 °C, followed by incubation with Alexa Fluor 488‐conjugated goat anti‐mouse IgG (Invitrogen, A‐11001) in PBS for 2 h at RT. Finally, samples were counterstained with 40,6‐diamidino‐2‐phenylindole (DAPI) and visualized with a laser scanning confocal microscope (LSM710, Zeiss, Germany).

Frozen sections were analyzed by histological H&E, Von Kossa, and Alizarin Red staining (Beijing Leagene Biotechnology, China), which were performed following the manufacturer's instructions.

For immunofluorescence staining, standard procedures were followed as previously described. Frozen sections were incubated with 5% normal goat serum (Life Technologies, 01‐6201) for 40 min at RT. For intracellular antigen staining, 0.1% Triton‐PBS was used to permeate the membrane before incubation with serum. Primary antibodies against MYC (Abcam, ab632), CD31 (BD Biosciences, 553370; Abcam, ab64543), eNOS (ABclonal, A15075), CD144 (Santa Cruz, sc‐9989), α‐SMA (Abcam, ab7817; ab5694), GFP (Abcam, ab6556), RFP (Invitrogen, MA5‐15257), CD45 (Abclonal, A2115; R&D, MAB114), Sca‐1 (Millipore, AB4336), Osteocalcin (Abcam, ab13420; Santa Cruz, sc‐365797), iNOS (Abcam, ab178945) and CD206 (Abcam, ab64693) diluted in PBS were then dropped to cover sections and incubated overnight at 4 °C. Then the sections were washed five times with PBS, followed by incubating with secondary antibody in PBS for 2 h at RT. All secondary antibodies were obtained from Invitrogen. The sections incubated with PBS were used as negative controls. Images were acquired under an advanced forward epifluorescence microscope (Ax10 Imager M2, Zeiss, Germany) or a laser scanning confocal microscope (LSM710, Zeiss, Germany), and quantified by Image J software.

### Quantification and Statistical Analysis

Data are expressed as mean ± standard error of the mean (SEM). GraphPad Prism Software Version 7.0 (San Diego, CA, USA) was used for statistical analysis. Comparisons of two groups were made with two‐tailed Student's *t*‐test. Comparisons among more than two groups were made by one‐way or two‐way analysis of variance (ANOVA) followed by Bonferroni's test for multiple pairwise comparisons. *p* < 0.05 was considered significant.

## Conflict of Interest

The authors declare no conflict of interest.

## Author Contributions

M.X. and F.W. equally contributed to this study. Q.Z. and G.D. conceived the original concept and initiated this project. Q.Z. designed the experiment and supervised the entire project. F.W. and M.Q. synthesized all compounds and prepared hydrogels. M.X., F.W., R.C., and H.W. performed the in vivo and in vitro experiments. M.X. and F.W. analyzed data under the supervision of Q.Z., Y.S., and H.J. helped with data collection. Q.Z., A.C.M., and G.D. supervised the work. M.X., A.C.M., and Q.Z. wrote and edited the manuscript with input from other authors.

## Supporting information



Supporting Information

## Data Availability

The data that support the findings of this study are available from the corresponding author upon reasonable request.
