# Supplementary material for: Localized COUP‐TFII pDNA Delivery Modulates Stem/Progenitor Cell Differentiation to Enhance Endothelialization and Inhibit Calcification of Decellularized Allografts
Source: Adv Sci (Weinh). 2024 Dec 10;12(5):2409744. doi: 10.1002/advs.202409744 (PMC11792037; doi:10.1002/advs.202409744)
Supplement: Supplementary file 1 — Supporting Information [file ADVS-12-2409744-s001.pdf]

ADVANCED SCIENCE
Open Access

Supporting Information

for *Adv. Sci.*, DOI 10.1002/advs.202409744

Localized COUP-TFII pDNA Delivery Modulates Stem/Progenitor Cell Differentiation to Enhance Endothelialization and Inhibit Calcification of Decellularized Allografts

Mengmeng Xing, Fei Wang, Ruowen Chu, He Wang, Yuyao Sun, Meng Qian, Huan Jiang, Adam C. Midgley, Guohao Dai* and Qiang Zhao**

Supporting Information

Localized COUP-TFII pDNA delivery modulates stem/progenitor cell differentiation to enhance endothelialization and inhibit calcification of decellularized allografts

Mengmeng Xing, Fei Wang, Ruowen Chu, He Wang, Yuyao Sun, Meng Qian, Huan Jiang, Adam C. Midgley, Guohao Dai*, Qiang Zhao**

# M. Xing and F. Wang equally contributed to this study.

M. Xing, F. Wang, R. Chu, H. Wang, Y. Sun, M. Qian, H. Jiang, Dr A.C. Midgley, Prof Q. Zhao
State key Laboratory of Medicinal Chemical Biology, Frontiers Science Center for Cell Responses, Key Laboratory of Bioactive Materials (Ministry of Education), College of Life Sciences, Nankai University, Tianjin 300071, China
E-mail: qiangzhao@nankai.edu.cn (Q. Zhao), midgleyac@nankai.edu.cn (A.C. Midgley)

Prof G. Dai
Department of Bioengineering, Northeastern University, Boston, Massachusetts 02115, USA
E-mail: g.dai@northeastern.edu

Prof Q. Zhao
The Institute of Cardiovascular Sciences, School of Basic Medical Sciences, State Key Laboratory of Vascular Homeostasis and Remodeling, Health Science Center, Peking University, Beijing 100191, China.

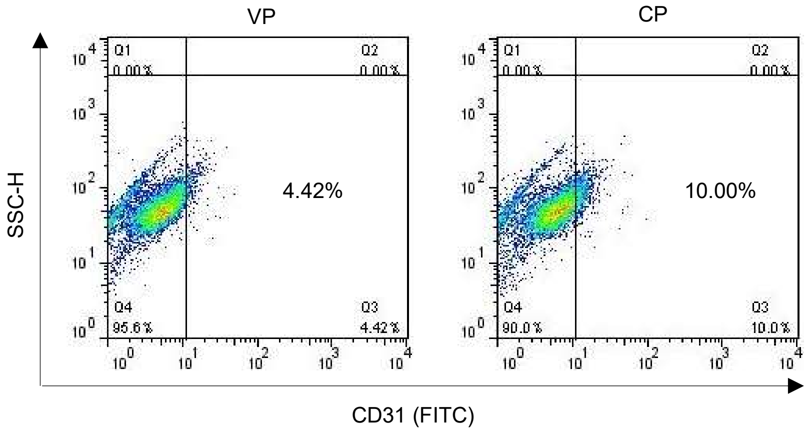

Figure S1. Flow cytometry analysis shows the differentiation of the SPCs overexpressed COUP-TFII.

A

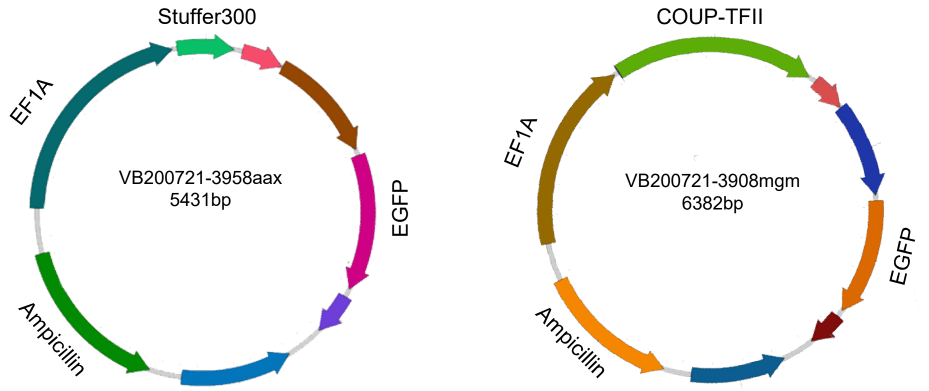

B

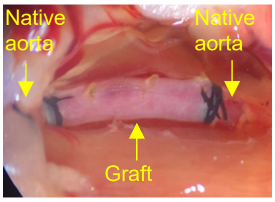

C

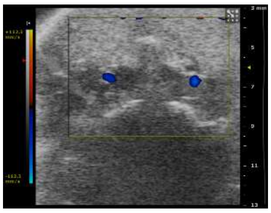

D

Patency rate	VP	CP
1 month	8/8	8/8
3 months	7/7	7/7

E

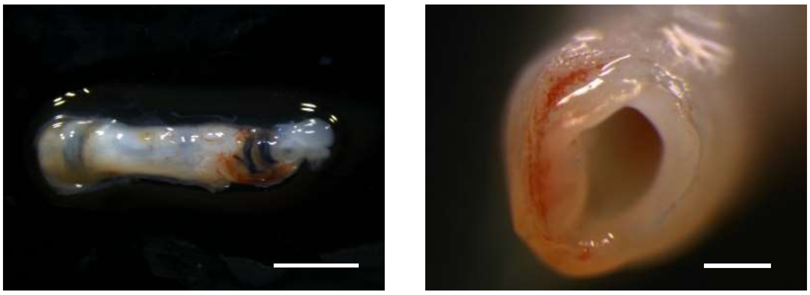

Figure S2. (A) Shown is the map of pDNA used in mouse carotid artery replacement model. **(B)** Representative image shows the graft after implantation. **(C)** Ultrasound image of the implanted graft is shown. **(D)** Statistic analysis of the patency rates of implanted vascular grafts. **(E)** Stereoscopic images shows the grafts explanted at 1 month. Scale bars: 2 mm (Left) or 500 μm (Right).

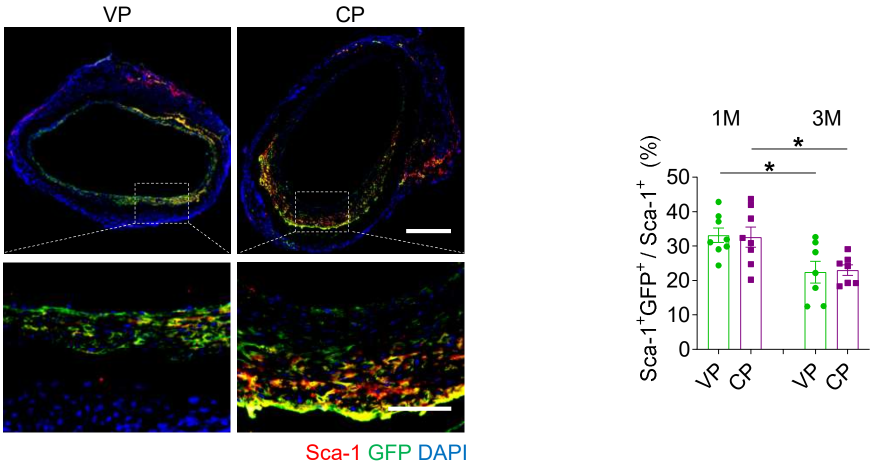

Figure S3. Shown is co-immunofluorescence staining for Sca-1 and GFP and quantification of the transfection efficiency (n=8 for 1 month; n=7 for 3 months). Scale bar: 100 μm. All data are presented as the means ± SEM. *: $p < 0.05$.

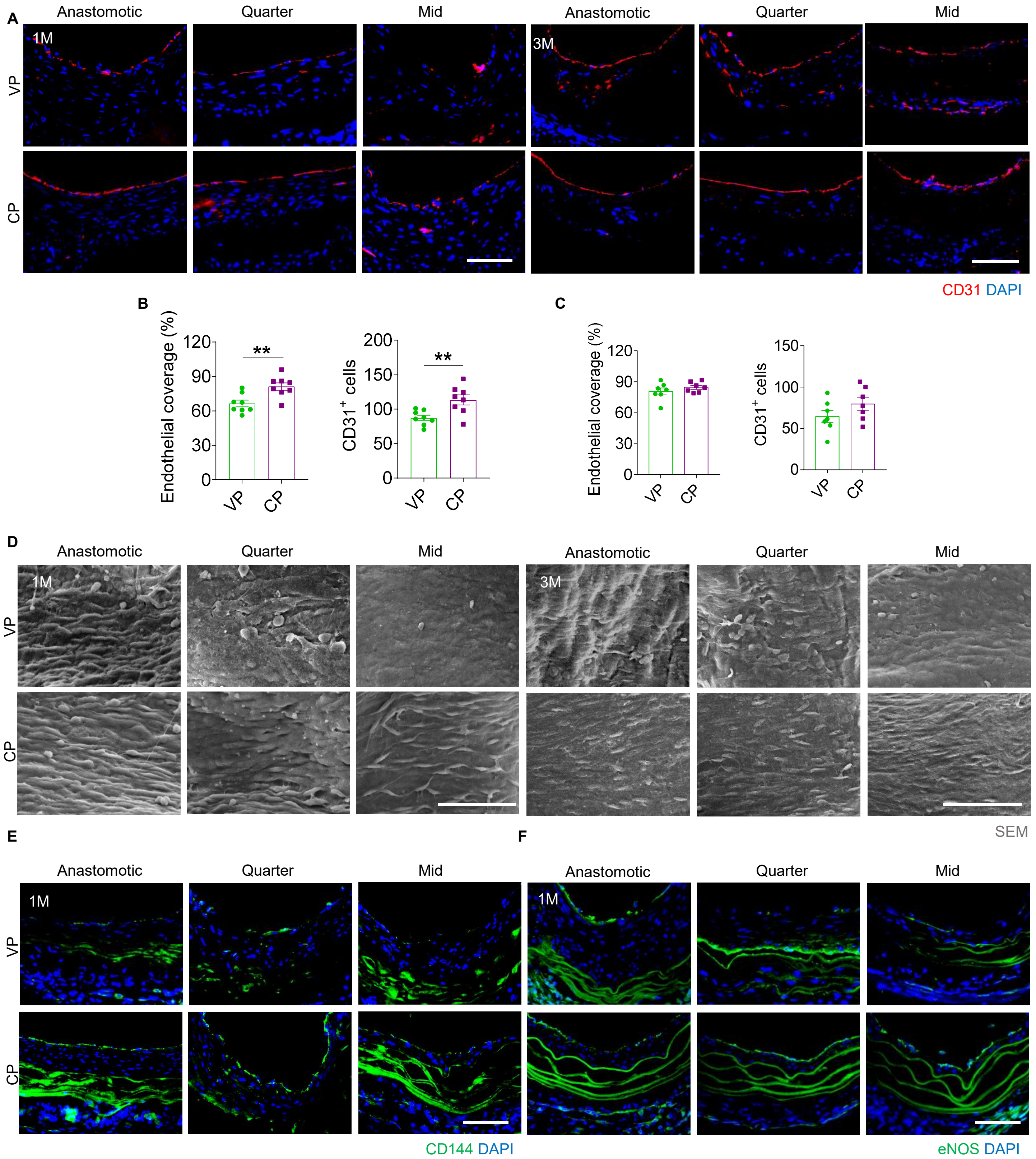

Figure S4. (A) Immunofluorescence staining for CD31 of vascular grafts is shown. Scale bar: 50 μm. **(B,C)** The endothelial coverage fraction and the number of CD31⁺ cells at 1 and 3 months were quantified, respectively (n=8 for 1 month, n=7 for 3 months). **(D)** SEM images shows the spindle ECs lining on the lumen of grafts. Scale bar: 50 μm. **(E)** Shown is immunofluorescence staining for CD144 of vascular grafts. Scale bar: 50 μm. **(F)** Immunofluorescence staining for eNOS of the explanted grafts is shown. Scale bar: 50 μm. All data are presented as the means ± SEM. **: $p < 0.01$.

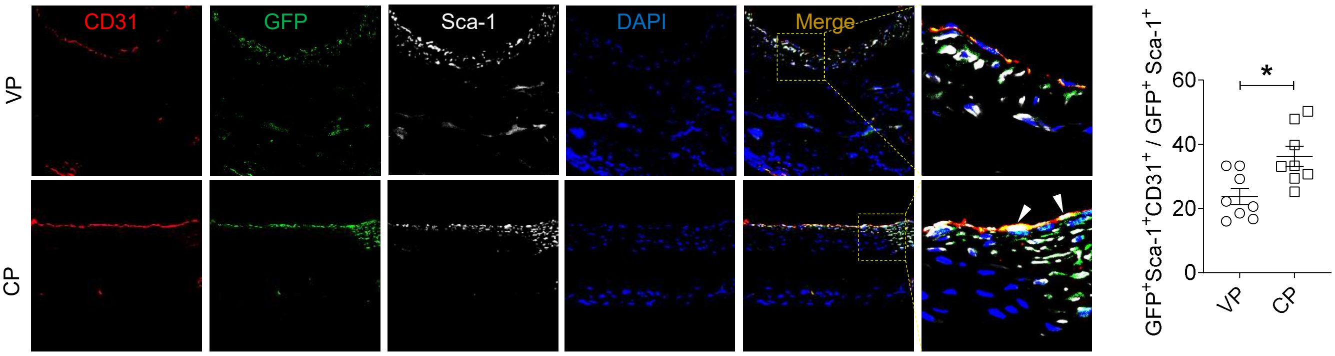

Figure S5. Shown is co-immunofluorescence staining for CD31, GFP and Sca-1 of the explanted grafts at 1 month. Scale bar: 50 μm. White arrows indicate the differentiated SPCs. The ratio of $GFP^{+}Sca\text{-}1^{+}CD31^{+}$ to $GFP^{+}Sca\text{-}1^{+}$ cells was further quantified (n=8). All data are presented as the means ± SEM. *: $p < 0.05$.

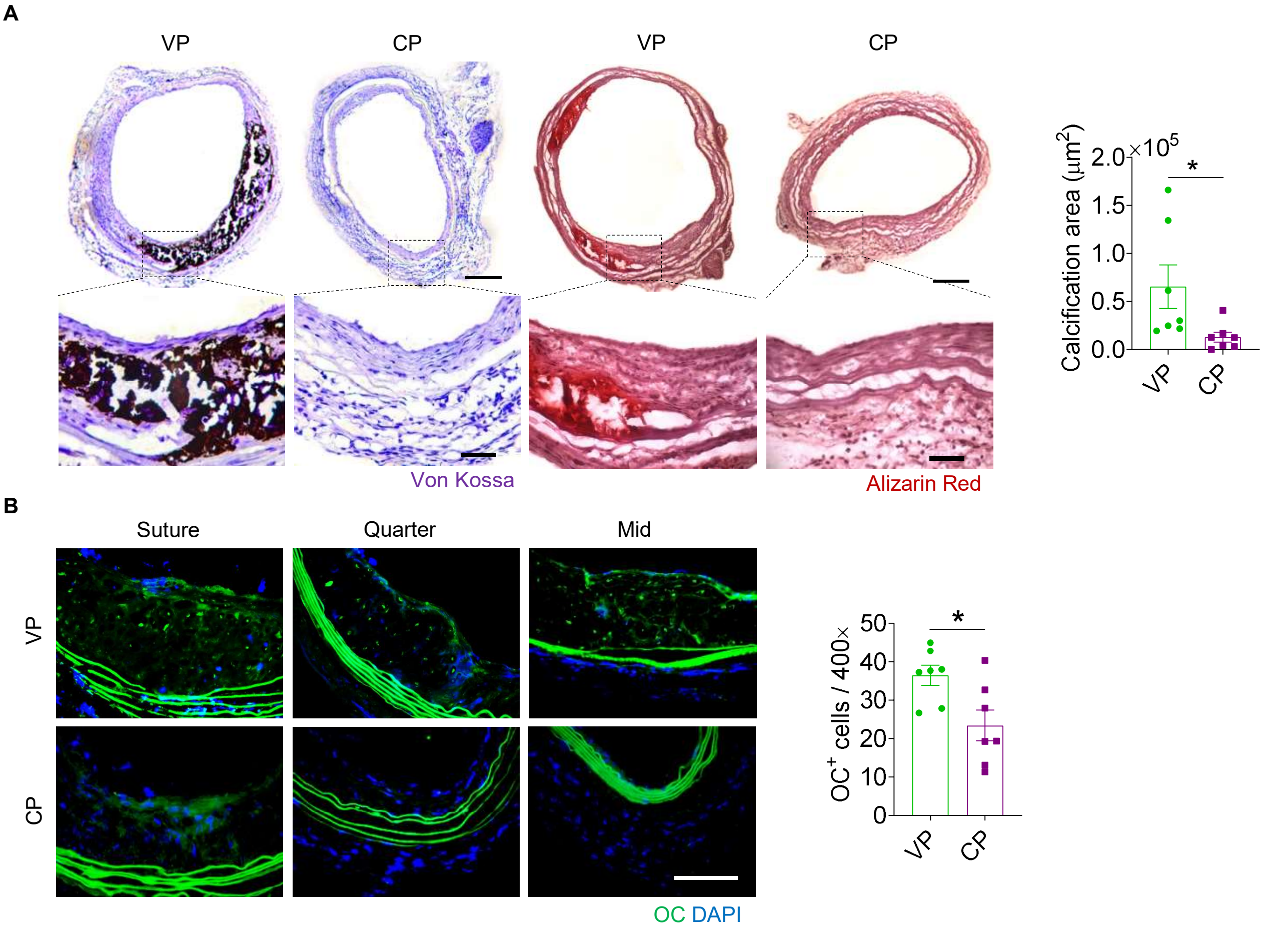

Figure S6. (A) Von Kossa and Alizarin Red staining of grafts explanted at 3 months are shown, and the calcification area was quantified according to Von Kossa staining (n=7). Scale bars: 200 μm or 50 μm (magnified images). **(B)** Shown is immunofluorescence staining for OC of grafts explanted at 3 months and quantification of the number of OC^+ cells (n=7). Scale bar: 50 μm. All data are presented as the means ± SEM. *: $p < 0.05$.

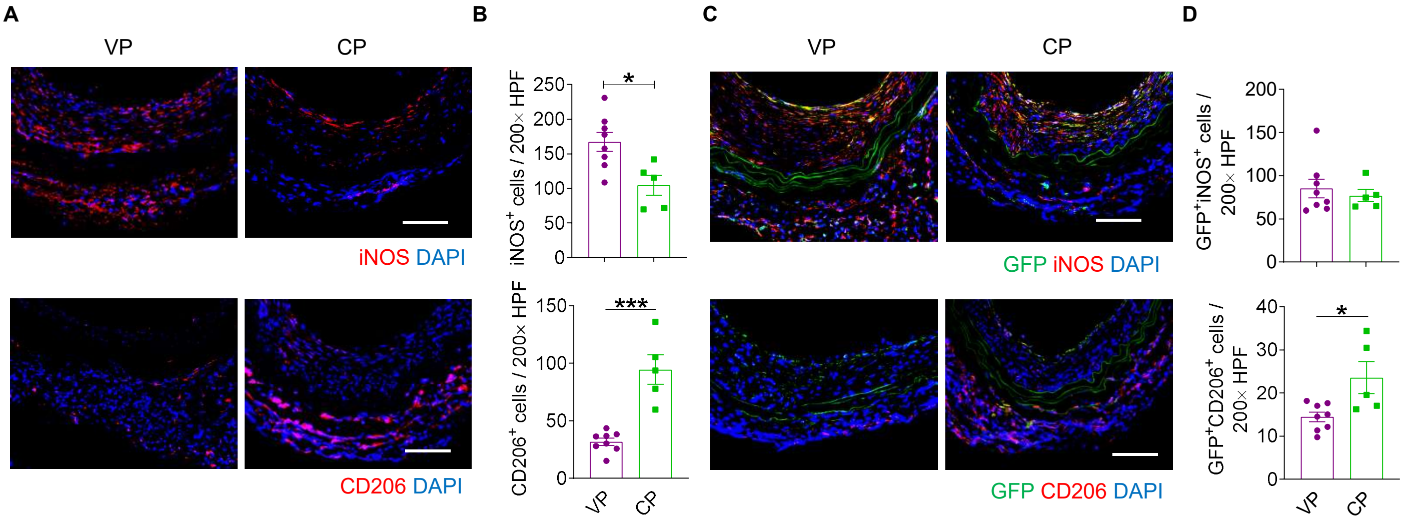

Figure S7. (A) Immunofluorescence staining for iNOS and CD206 of grafts explanted at 1 month are shown. Scale bar: 100 μm. **(B)**The number of $iNOS^+$ and $CD206^+$ cells were quantified (n=8). **(C)** Co-immunofluorescence staining for GFP and iNOS, GFP and CD206 of grafts explanted at 1 month are shown. Scale bar: 100μm. **(D)** The number of GFP^+iNOS^+ cells and GFP^+CD206^+ cells were quantified (n=8). All data are presented as the means ± SEM. *: $p < 0.05$, ***: $p < 0.001$.

A

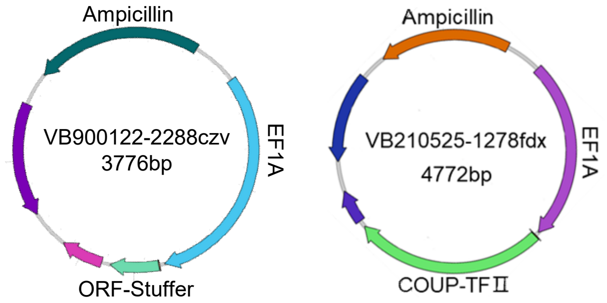

B

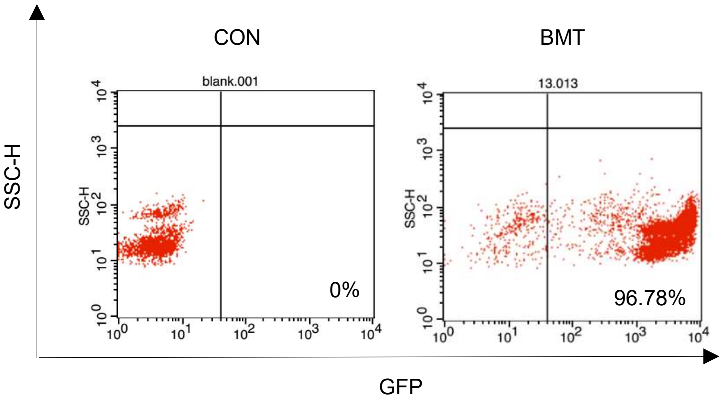

Figure S8. (A) Shown is the map of pDNA used in reconstituted bone marrow chimera mouse carotid artery replacement model. **(B)** Flow cytometry analysis shows the blood reconstruction efficiency after bone marrow transplantation.

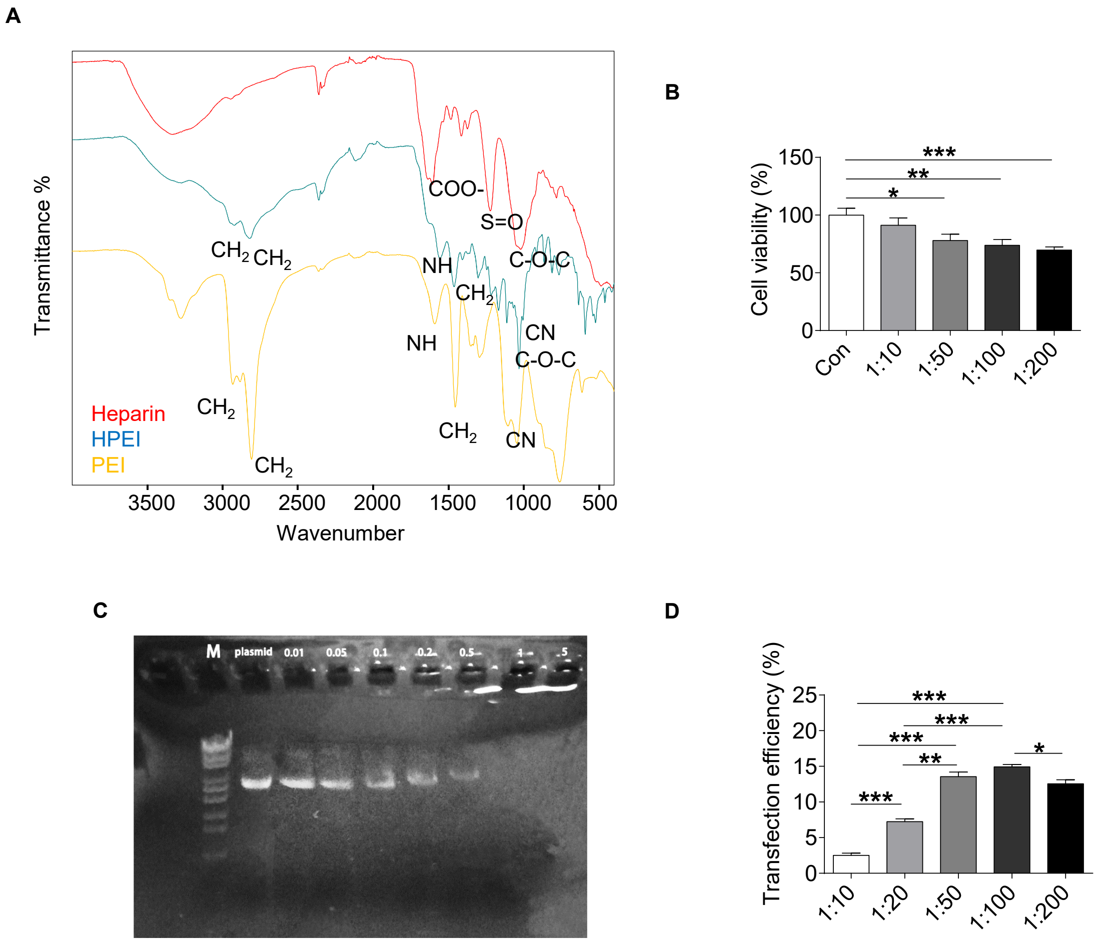

Figure S9. (A) Infrared spectroscopy of the heparin, PEI and HPEI nanoparticles is shown. **(B)** CCK8 assay shows the cytotoxicity of HPEI nanoparticles. **(C)** Shown is the ability of the HPEI nanoparticles absorb plasmid DNA. **(D)** The transfection efficiency was quantified by flow cytometry. All data are presented as the means ± SEM. *: $p < 0.05$, **: $p < 0.01$, ***: $p < 0.001$.

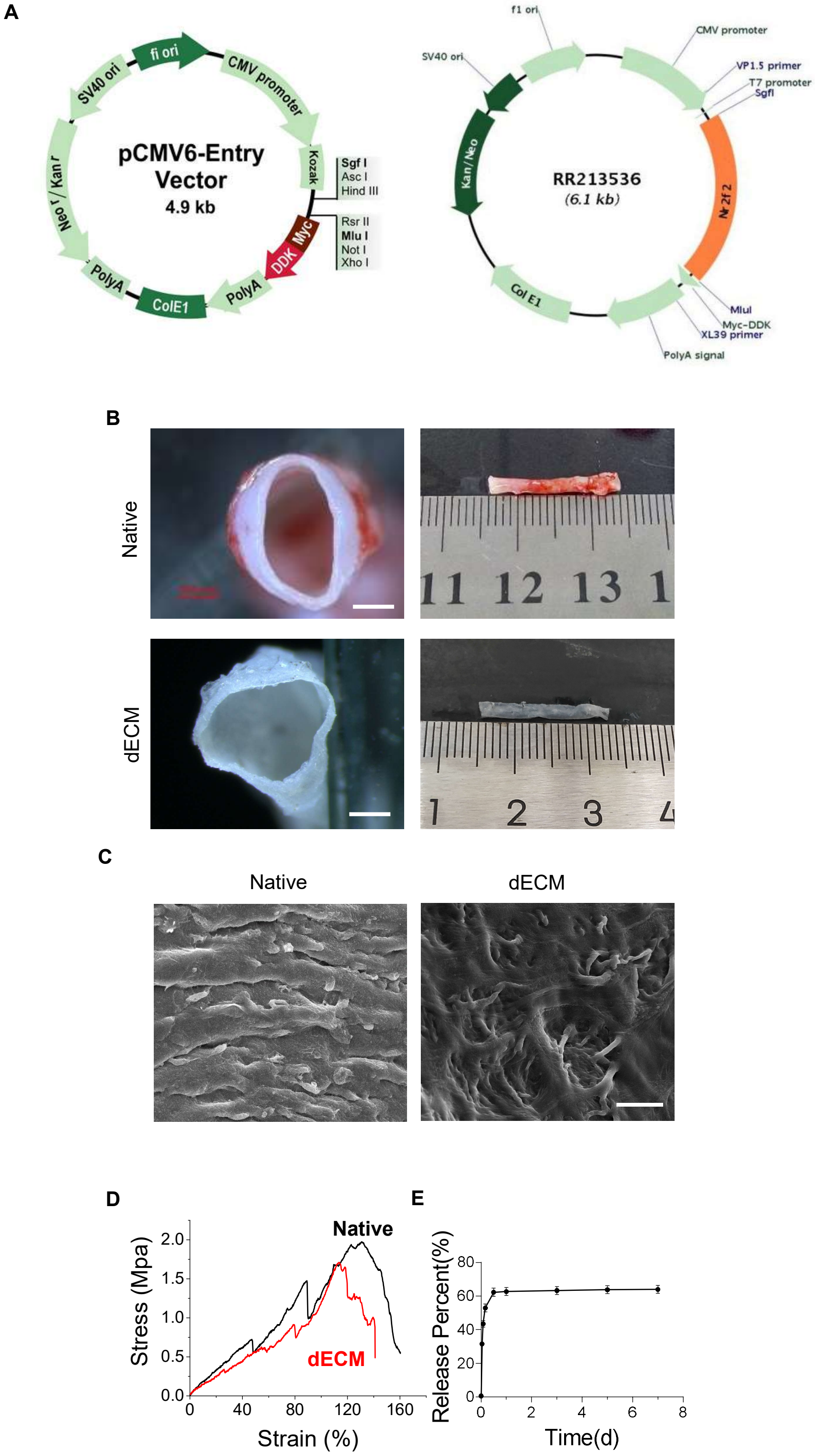

Figure S10. (A) Shown is the map of plasmids used in rat abdominal artery replacement model. (B) Stereoscopic images show the allograft before and after decellularization. Scale bar: 500 μm. (C) The SEM images of the allografts are shown. Scale bar: 20 μm. (D) Representative stress-strain curves are shown of the allograft before and after decellularization (n=3). (E) *In vitro* release of the COUP-TFII@HPEI nanoparticles from the decellularized allograft is shown (n=3).

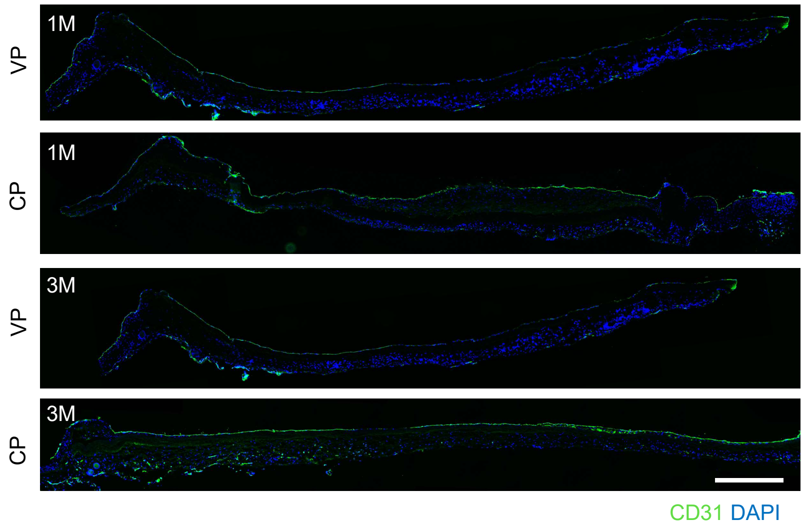

Figure S11. Immunofluorescence staining of longitudinal section is shown for CD31. Scale bar: 50 µm.

Table S1. Real-time qPCR primer sequences.

Genes	species	Forward primer	Reverse primer
Nr2f2	rats	5’-TCAGATGCCTGTGGTCTGTC-3’	5’-AAAGCTTTCCGAACCGTGT-3’
Pecam1	rats	5’-CTGGGAGGTATCGAATGGGC-3’	5’-GTGCATTTGTACTTCCCGGC-3’
Tek	rats	5’-AAGAGCGAGTAGACCATGCG-3’	5’-ACTAGTCCATAAAGGAGCAAGC-3’
Angpt1	rats	5’-AGCATGTGATGGAAAATTATACT-3’	5’-AGTACCTGGGTCTCCACATC-3’
Acta2	rats	5’-CTGCCTTGGTGTGTGACAATGG-3’	5’-CGGGTACTTCAGGGTCAGGATTC-3’
Cnn1	rats	5’-ACCAAGCGGCAGATCTTTGA-3’	5’-CATCTGCAAGCTGACGTTGA-3’
Tagln	rats	5’-TTCTGCCTCAACATGGCCAAC-3’	5’-CACCTTCACTGGCTTGGATC-3’
Myh11	rats	5’-GCACAAGAAGAAGAAGCTGGA-3’	5’-TTGAGCATGCCTGTGACACT-3’
Gapdh	rats	5’-GTCGGTGTGAACGGATTTG-3’	5’-TCCCATTCTCAGCCTTGAC-3’
